# Systems biology reveals how altered TGFβ signalling with age reduces protection against pro-inflammatory stimuli

**DOI:** 10.1371/journal.pcbi.1006685

**Published:** 2019-01-24

**Authors:** David Hodgson, Andrew D. Rowan, Francesco Falciani, Carole J. Proctor

**Affiliations:** 1 Institute of Cellular Medicine, Ageing Research Laboratories, Campus for Ageing and Vitality, Newcastle University, Newcastle upon Tyne, United Kingdom; 2 MRC/Arthritis Research UK Centre for Musculoskeletal Ageing (CIMA), United Kingdom; 3 Skeletal Research Group, Institute of Genetic Medicine, Newcastle University, Newcastle upon Tyne, United Kingdom; 4 Institute of Integrative Biology, University of Liverpool, Liverpool, United Kingdom; University of Virginia, UNITED STATES

## Abstract

Osteoarthritis (OA) is a degenerative condition caused by dysregulation of multiple molecular signalling pathways. Such dysregulation results in damage to cartilage, a smooth and protective tissue that enables low friction articulation of synovial joints. Matrix metalloproteinases (MMPs), especially MMP-13, are key enzymes in the cleavage of type II collagen which is a vital component for cartilage integrity. Transforming growth factor beta (TGFβ) can protect against pro-inflammatory cytokine-mediated MMP expression. With age there is a change in the ratio of two TGFβ type I receptors (Alk1/Alk5), a shift that results in TGFβ losing its protective role in cartilage homeostasis. Instead, TGFβ promotes cartilage degradation which correlates with the spontaneous development of OA in murine models. However, the mechanism by which TGFβ protects against pro-inflammatory responses and how this changes with age has not been extensively studied. As TGFβ signalling is complex, we used systems biology to combine experimental and computational outputs to examine how the system changes with age. Experiments showed that the repressive effect of TGFβ on chondrocytes treated with a pro-inflammatory stimulus required Alk5. Computational modelling revealed two independent mechanisms were needed to explain the crosstalk between TGFβ and pro-inflammatory signalling pathways. A novel meta-analysis of microarray data from OA patient tissue was used to create a Cytoscape network representative of human OA and revealed the importance of inflammation. Combining the modelled genes with the microarray network provided a global overview into the crosstalk between the different signalling pathways involved in OA development. Our results provide further insights into the mechanisms that cause TGFβ signalling to change from a protective to a detrimental pathway in cartilage with ageing. Moreover, such a systems biology approach may enable restoration of the protective role of TGFβ as a potential therapy to prevent age-related loss of cartilage and the development of OA.

## Author summary

Osteoarthritis (OA) is a debilitating disease that is a consequence of cartilage degeneration, often for a variety of reasons. Age is typically the driving force behind OA, and one reason is that biological pathways change as we get older and can become damaging. Transforming growth factor beta (TGFβ) is one such pathway, which in young tissues has an important role in keeping the joint healthy as well as protecting against damage caused by inflammation. As we age this pathway starts to promote damage. However, how its effect on inflammation changes has not yet been studied in detail. We used a range of experimental and computational techniques to explore how TGFβ helps to protect against inflammation, but also how these interactions change as we age. We found that with ageing TGFβ may lead to a prolonged inflammatory response in older individuals, resulting in more damage than it normally would in a younger individual suggesting a once protective pathway can now lead to prolonged damage. A better understanding of this change may allow us to develop drugs to repair this pathway, and re-establish its once protective effect.

## Introduction

Osteoarthritis (OA) is a spectrum of degenerative disorders that become much more prevalent with age to the extent that 50% of those aged ≥65 years suffer from the disease globally [1]. Treatments are limited with no therapies that directly target the disease; instead, the focus is on relieving symptoms and improving function. This is achieved through the use of painkilling medications in combination with physiotherapy, as well as educating patients to manage their weight, with the last resort being total joint replacement [2]. Understanding where and when to target OA progression is complex due to the multifactorial nature of OA development. For example, many pathways such as Wnt/β-catenin, mTor, phosphatidylinositol 3-kinases(PI3K)/Akt, Indian Hedgehog, protein kinase K and Notch have been linked to the destruction of cartilage and the development of OA based on animal model data [3–8].

Transforming growth factor β (TGFβ) is a growth factor with significant therapeutic potential due to its pleiotropic role in disease progression. It has varying effects depending on not only the type of tissue but the age and environment surrounding the tissue. TGFβ activates signalling pathways that typically have anabolic effects on cartilage such that targeting components of this pathway has potential for new treatment therapies. For example, it has been shown that TGFβ is required for normal cartilage development and is crucial for maintaining chondrocyte homeostasis in synovial joints [9]. In addition, we and others have reported that TGFβ has a protective effect against a multitude of inflammatory stimuli [10–12], including interleukin-1 alpha (IL-1α, henceforth referred to as IL-1) in combination with oncostatin M (OSM) (cytokines known to be elevated in OA synovial fluid which markedly induce cartilage destruction [13]). This potent inflammatory stimulus promotes the expression of the collagenases, matrix metalloproteinase (*MMP*)*1* and *MMP13*, whilst supressing the expression of their endogenous inhibitor, tissue inhibitor of metalloproteinases (*TIMP*)*1* [14]. Inclusion of TGFβ has been shown to reverse this expression profile [10]. IL-1+OSM has been shown to activate multiple signalling pathways in chondrocytes [15–17], but the mechanism by which TGFβ blocks such cytokine-induced MMP expression in human chondrocytes has not been described. TGFβ is known to have a role in matrix production by inducing collagen types I and II, and proteoglycans [18]. It also counteracts major catabolic genes such as Runt-related transcription factor 2 (*RUNX2*), a disintegrin and metalloproteinase with thrombospondin motifs 5 (*ADAMTS5*) and collagen 10 [19], as well as inducing TIMPs to inhibit metalloproteinase-mediated damage [20].

The canonical TGFβ pathway can have either catabolic or anabolic effects depending on which of its receptors is activated [21]. In healthy cartilage, TGFβ signals through two type 1 TGF receptors, activin-like kinase 1 and 5 (Alk1 (*ACVRL1*) and Alk5 (*TGFBR1*), respectively). TGFβ predominantly signals through Alk5 due to its abundance [21]. Alk1 and Alk5 both form dimers to mediate their effect. Alk5 forms homodimers that in turn bind TGFβ, whilst Alk1 forms a heterodimer with Alk5 such that Alk1 signalling is dependent on Alk5 [22]. Alk5 binding to TGFβ leads to Smad 2/3 phosphorylation, which blocks terminal differentiation, chondrocyte hypertrophy and also stimulates matrix production [21]. When the balance shifts toward Alk1 signalling, this causes increased Smad 1/5/8 phosphorylation [21] which enhances chondrocyte hypertrophy, terminal differentiation and matrix breakdown by MMPs such as MMP-13 [21].

Age is believed to be the major driving factor for conversion of Alk5 to Alk1 signalling in cartilage, with Alk5 levels decreasing more rapidly than Alk1 leading to an increase in the Alk1/Alk5 ratio [21]. It has been suggested that Alk5 levels may decrease due to a change in the ratio of Alk5 synthesis/degradation with age [23], but the mechanisms involved are unknown. Of particular interest is how a change in the Alk1/Alk5 ratio affects the ability of TGFβ to protect against the damaging effects of inflammatory insults such as IL1+OSM.

The construction, and development, of computational models has previously aided in our understanding of TGFβ signalling [24]. In order to better understand TGFβ interactions with pro-inflammatory stimuli, we modified and combined two previously published models [23,25] to provide new insight into the complex interactions that allow TGFβ to mediate its protective effects against inflammation, as well as how these interactions change with ageing. To contextualise this model in a global environment, we examined publically available microarray data to reveal how dysregulation of one system can affect a variety of others, ultimately leading to OA progression.

## Results

### TGFβ represses the induction of *MMP13* expression by IL-1+OSM in chondrocytes via Alk5 signalling

**Fig 1:**
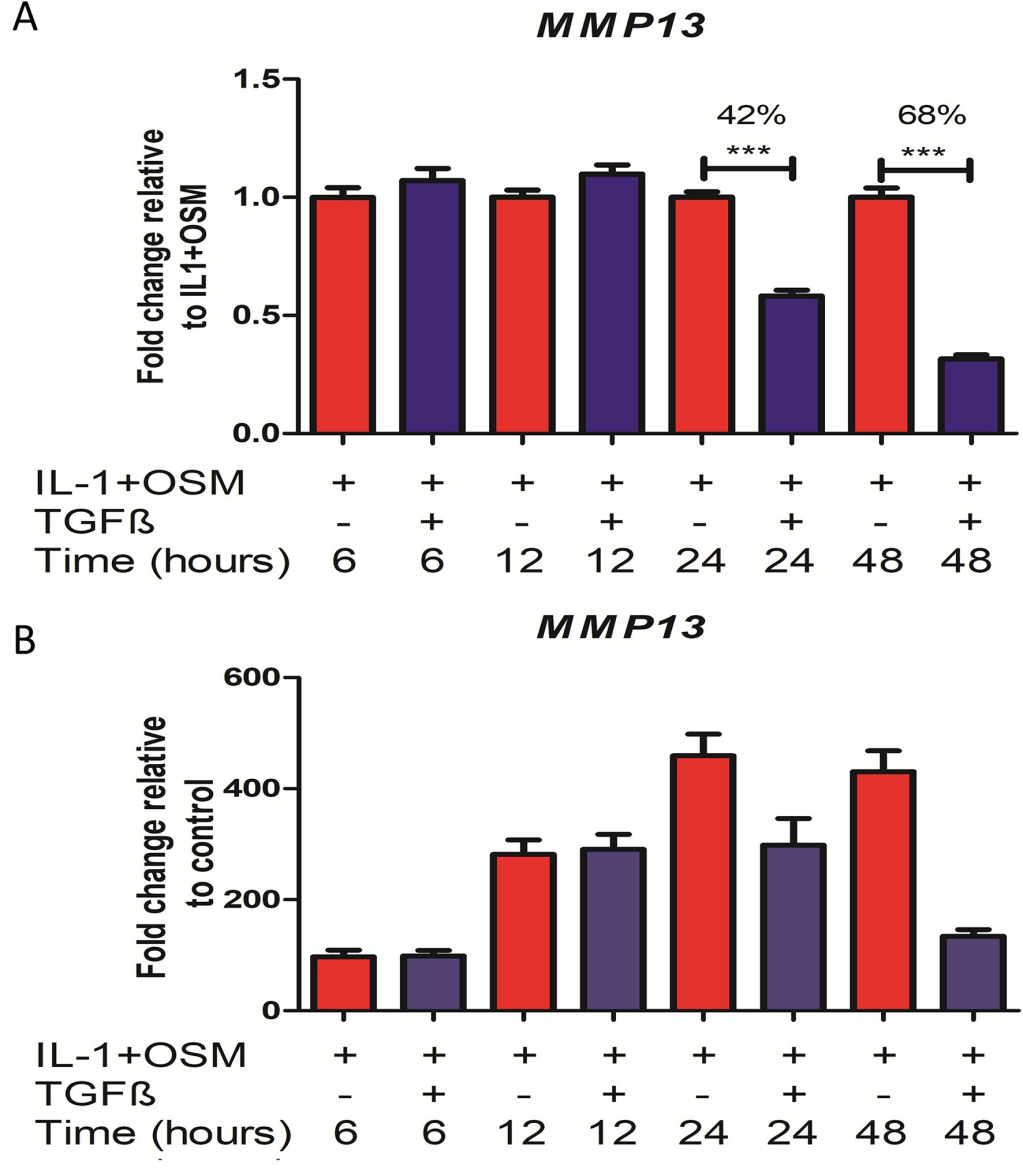
Effect of TGFβ on IL1+OSM-induced *MMP13* expression. SW1353 chondrocytes were stimulated with IL-1 (0.5 ng/ml) + OSM (10 ng/ml) ± TGFβ (10 ng/ml) for the indicated durations. qPCR was then performed on isolated mRNA to measure *MMP13* expression whilst glyceraldehyde 3-phosphate dehydrogenase (*GAPDH*) was used for normalisation purposes. (A) Data are presented as fold change relative to IL-1+OSM (normalized to 1.0 for each time point; mean ± SEM). (B) Data are presented as fold change relative to control. Data are pooled from at least 4 independent experiments (n ≥ 22) and statistical comparisons performed using an unpaired Student’s t-test, where ***p < 0.001 vs IL-1+OSM. Data used to construct figure are provided in S1 File.

We used an experimental approach to examine how TGFβ represses the induction of *MMP13* by a pro-inflammatory stimulus. We first confirmed the previously shown synergistic effect of IL-1+OSM on *MMP13* induction [13,26] in SW1353 chondrocytes could be repressed by TGFβ (at varying doses) as we have previously reported in both human and bovine cartilage [10,27,28] (see S1 Fig). This repression took longer than 12 hours, becoming evident at 24 hours and increasingly potent with time: combined data from multiple cell populations indicated the repression was approximately 42% of the IL-1+OSM induction at 24 hours, rising to 68% at 48 hours (Fig 1A). Note that the dose of IL-1 used was quite low (0.5 ng/ml) as previous studies have shown that low concentrations of IL-1 have a biological effect in SW1353 cells [29]. In addition, it should be pointed out that Fig 1 has unusually high n values. This is because we needed an accurate determination of the percentage of repression for constructing the model. We also showed the kinetics of *MMP13* induction by IL1+OSM with and without TGFβ by normalising the data at each time point to the control values (Fig 1B). This shows that in the absence of TGFβ *MMP13* increases up to 24 hours and then starts to decline at 48 hours. The addition of TGFβ does not alter the kinetics for the first 12 hours but prevents any further increase at 24 hours and by 48 hours *MMP13* levels are greatly reduced. It is important to note that *MMP13* is nearly absent in untreated controls, this results in a high CT value. Therefore, any change in the basal expression can result in vastly different fold change values for the treated samples (Fig 1B). The repressive effect seen by TGFβ was more constant across samples (Fig 1A).

Treatment of SW1353 chondrocytes with TGFβ (2 h) led to a significant upregulation of the Alk5-inducible gene plasminogen activator inhibitor-1 (*PAI1*) [30], whilst the Alk1-dependent gene inhibitor of DNA binding 1 (*ID1*) [31] was unaffected (S2 Fig). In line with this observation, assessment of receptor expression revealed *TGFBR1* (Alk5) expression was higher than *ACVRL1* (Alk1) (S3 Fig). We next confirmed that siRNA-mediated *TGFBR1* silencing (S4 Fig) prevented the observed repression of *MMP13* repression by TGFβ (S2 Fig).

### Computational modelling reveals two independent mechanisms are required for TGFβ-mediated repression of IL-1+OSM-induced *MMP13* expression

**Fig 2:**
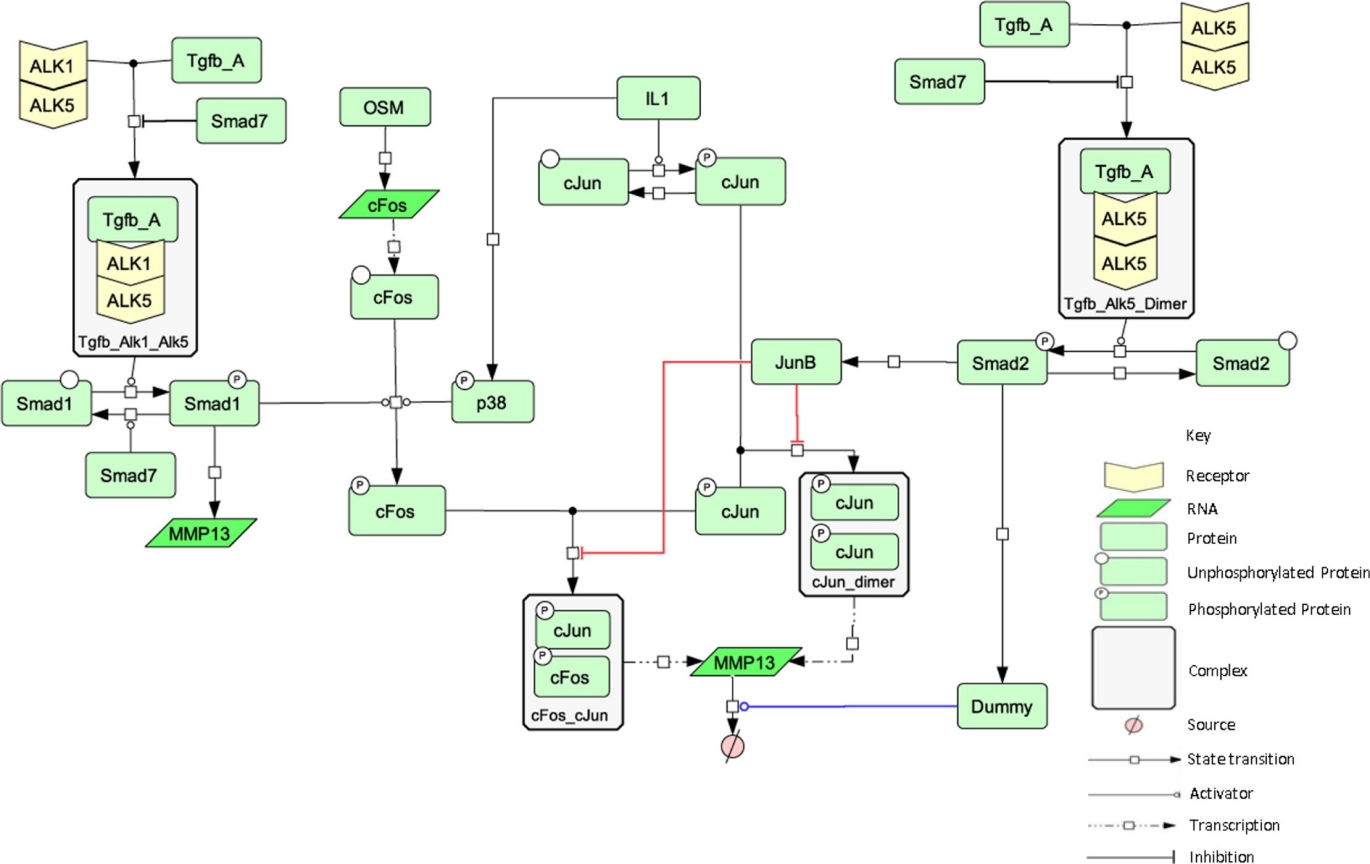
A simplified network diagram showing the key interactions between the IL-1, OSM and TGFβ components. The focus of this simplified schematic of the complete model is specifically on the key reactions that mediate the interactions between IL-1, OSM and TGFβ. The red lines highlight JunB interactions with the AP-1 complex and the blue line indicates the dummy species that helps drive *MMP13* mRNA degradation. A full schematic is shown in S5 Fig.

**Fig 3:**
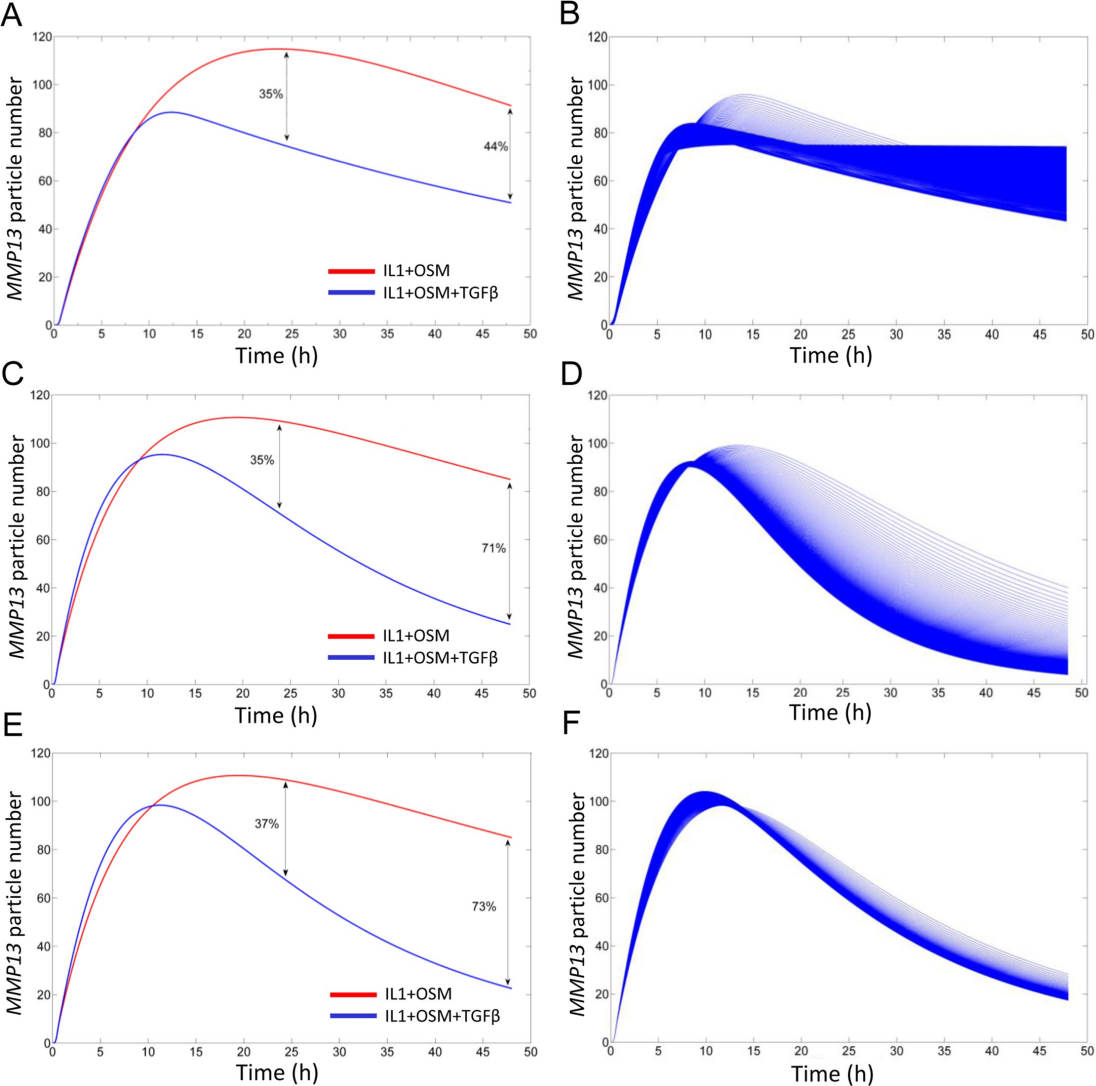
Simulating the TGFβ mediated repression of IL-1+OSM-driven *MMP13* expression. (A, C, E) Deterministic model run over a simulation time of 48 h. Initially, the total pool of TGFβ is active in the model to replicate experimental conditions. The red lines show IL-1+OSM-induced *MMP13* modelled in the absence of TGFβ whilst the blue lines show the model with TGFβ included. (A) Modelled with only the AP-1 interactions component. (C) Modelled with only the mRNA instability component. (E) Modelled with both AP-1 interactions and mRNA instability components. (B, D, F) Simulation results from a deterministic parameter scan changing the initial concentration of active TGFβ in the model by 1000-fold, and simulated over 48 h using COPASI. (B) Modelled with only the AP-1 interactions component. (D) Modelled with only mRNA instability component. (F) Modelled with both AP-1 interactions and mRNA instability components. Model parameters are provided in S1 and S2 Files.

Computational modelling was used to explore how TGFβ-mediated repression of IL-1+OSM-induced *MMP13* expression changed with age. Using the experimental data generated previously we combined, and then expanded upon, models of IL-1+OSM [25] and TGFβ [23] signalling in chondrocytes (see S2 File). In the combined model, we first assumed that TGFβ signalling through Alk5 could only impact *MMP13* expression via JunB interactions with activator protein-1 (AP-1) complexes that consist of either c-Jun/c-Fos heterodimers or c-Jun homodimers (Fig 2; reactions highlighted in red). With this assumption the model simulations matched the repression seen experimentally at 24 h, although it could not replicate the increased repression observed at 48 h (Fig 3A and 3B). We hypothesised that the enhanced repression at 48 h could be due to increased *MMP13* mRNA degradation (Fig 2; reaction highlighted in blue). As the protein(s) and/or microRNA(s) responsible for this are currently unknown, we included a dummy species to mediate this effect. With the model adapted to include increased *MMP13* mRNA degradation (instead of transcriptional repression via AP-1 interactions), the simulations were able to match the repression seen at both 24 and 48 h (Fig 3C). However, this model failed to fully replicate the minor effects that changes in TGFβ concentration had on repression we had observed experimentally (Fig 3D and S1 Fig). We next used a model that included both transcriptional repression via AP-1 interactions and increased degradation of *MMP13* mRNA (simplified and complete model schematics are presented in Fig 2 and S5 Fig, respectively) and could simulate comparable levels of repression to those observed experimentally (Fig 3E), with changes in TGFβ concentration having minimal effects on the simulated *MMP13* repression (Fig 3F). The model also closely matched the kinetics of *MMP13* induction after stimulation by IL1+OSM shown experimentally (Fig 1B).

### Increased TGFβ signalling through Alk1 with age alters the response to a pro-inflammatory stimulus

**Fig 4:**
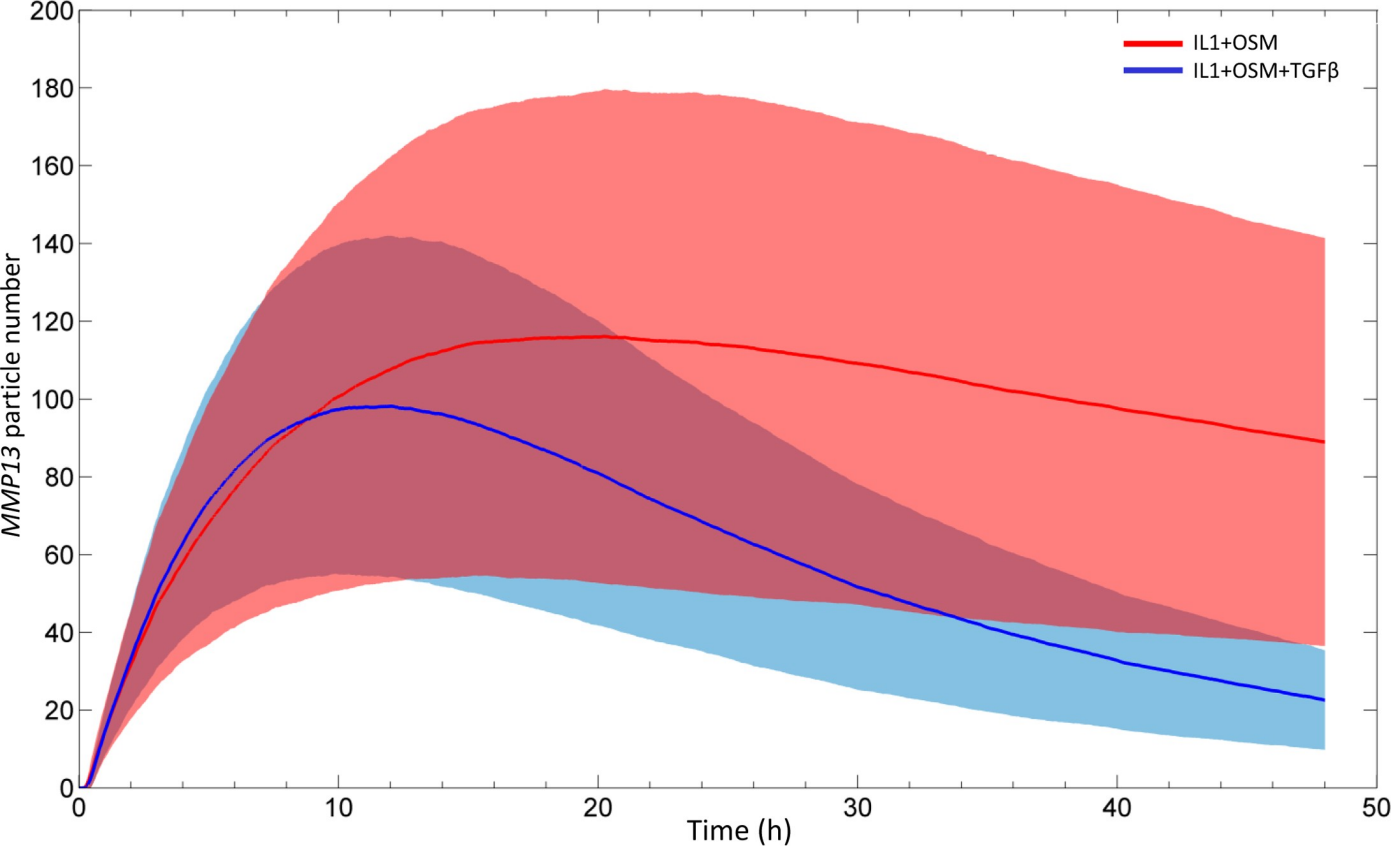
Average behaviour of 100 stochastic model runs showing inherent variation in the system. Stochastic simulation results showing the average behaviour ± the standard deviations of 100 stochastic runs of the complete model (Fig 2), run over a simulation time of 48 h. The model was run with IL-1+OSM (red line) or IL-1+OSM±TGFβ (blue line) where initially the total pool of TGFβ was in an active form. Curves show how the mean particle numbers of *MMP13* mRNA change over the simulation; the coloured shading shows the variation (one standard deviation above or below the mean) at each time point. Model parameters are provided in S1 and S2 Files.

We next evaluated the reliability of the predictions from our model by assessing the effect of a 6 h pre-treatment with TGF**β** on the IL-1+OSM response. The model predicted that *MMP13* repression would be observed after 12 hours of IL-1+OSM treatment, and this would subsequently increase; the predictions matched experimental data (see S6 Fig). Confident we could make accurate predictions with the model, we then simulated the model stochastically over 48 h. This predicted that the levels of *MMP13* mRNA generated by IL-1+OSM stimulation are highly variable whilst inclusion of TGF**β** reduced this variability from around 12 h onwards (Fig 4).

**Fig 5:**
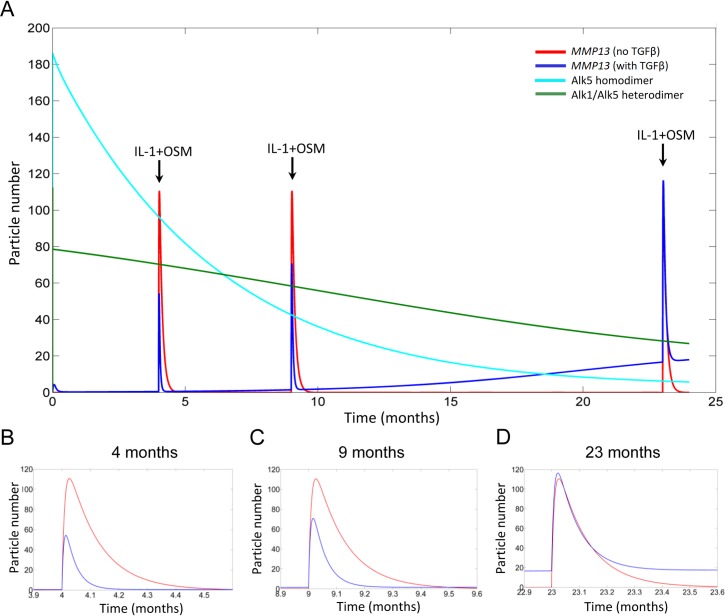
Modelling age-related changes of TGFβ responses to a pro-inflammatory stimulus. Deterministic simulation results for the model presented in Fig 2 run over a 24 month simulation time, using COPASI. The simulation was run with the total pool of TGFβ initially being in an inactive form to allow for the activation and deactivation of the growth factor throughout the simulation. IL-1+OSM were triggered using events at three time points at which the Alk1/Alk5 heterodimer:Alk5 homodimer ratio had changed. (A) Simulation output over 24 month period. Enlarged views of the change in *MMP13* mRNA expression immediately before and after IL-1+OSM stimulation events at 4 months (B), 9 months (C) and 23 months (D) are shown. Changes in Alk5 homodimers (light blue line), Alk1/Alk5 heterodimers (green line), *MMP13* mRNA expression in the absence of TGFβ (red line) or *MMP13* mRNA expression in the presence of TGFβ (dark blue line) over 24 months are presented. Model parameters are given in S1 and S2 Files.

A 24 month time simulation, representative of an ageing model, resulted in decreased Alk5 abundance including the availability of Alk5 homodimers (Fig 5A). Alk1/Alk5 heterodimers only declined slightly such that the Alk1/Alk5 ratio increased across the simulation, replicating what occurs with age [21]. We used the model to examine how ageing affects a pro-inflammatory stimulus (IL-1+OSM). At early time points (4 months) when Alk5 homodimers dominate, the pro-inflammatory stimulus was markedly abrogated by TGFβ with *MMP13* mRNA expression returning to basal levels rapidly (Fig 5B). The Alk1/Alk5 heterodimers and Alk5 homodimer ratios reached equality at about 7 months. Shortly after this (9 months), the *MMP13* repression was still evident albeit slightly reduced (Fig 5C). However, once Alk1 becomes the dominant receptor (e.g. 23 months), basal *MMP13* mRNA levels are higher in the presence of TGFβ resulting in a marginally higher level of *MMP13* mRNA following IL-1+OSM addition (Fig 5D); thus, the overall increase in *MMP13* from basal levels induced by IL-1+OSM addition was less compared to IL-1+OSM+TGFβ suggesting there was still some minor repression (Fig 5D). Furthermore, the induced *MMP13* persisted for longer compared to earlier time points, and returned to a basal expression level that was higher compared to time points when Alk5 was dominant (compare Fig 5B–5D).

### Model predicts that the effects of potential interventions depend on the Alk1/Alk5 ratio

An advantage of computational modelling is that it can be used to make predictions and test possible therapeutic interventions. In order to test the effect of ameliorating the damaging effects of TGFβ it was necessary to modify the model to allow for RUNX2 inactivation via a mechanism that was independent of TGFβ/Alk5 signalling. The previous assumption had been a simplification but it is known that other mechanisms are involved [32]. Therefore an additional reaction for RUNX2 inactivation was added to the model, although it had little effect on the model output in the presence of TGFβ (S7 Fig).

### Anti-TGFβ treatment

**Fig 6:**
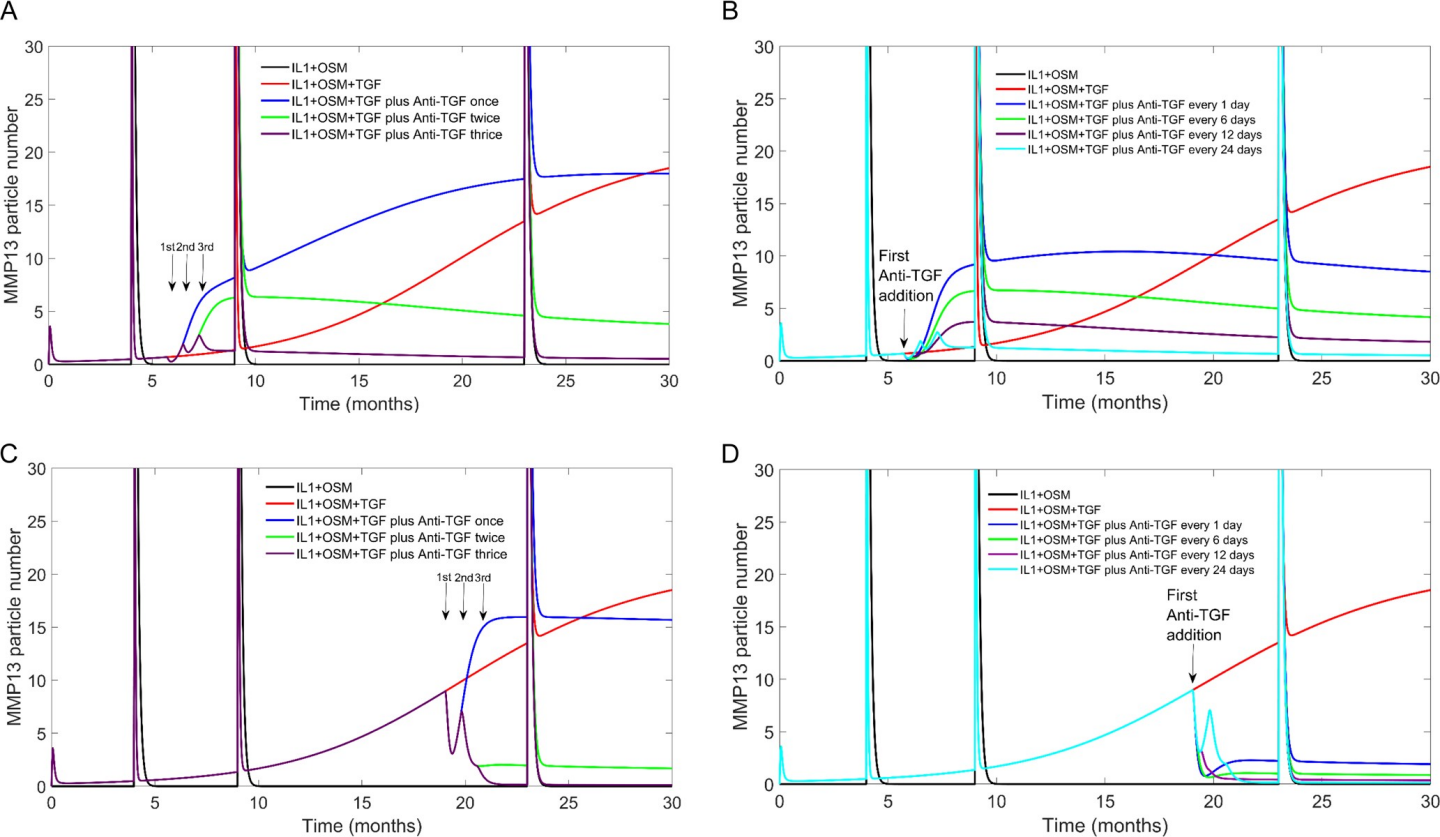
The effects of Anti-TGF treatment, on MMP-13 mRNA expression. Deterministic simulation results for the complete model, with an extra reaction to allow RUNX2 inactivation. The data show the change in MMP-13 mRNA across a 30-month simulation time. IL-1+OSM were triggered using events at 4, 9 and 23 months. The model also contained the species “Anti-TGF” which targeted and degraded both active and inactive TGFβ. Anti-TGF concentration was at zero when the simulation started. A) At 6 months Anti-TGF was added to the system, using an event. Anti-TGF was added either once, twice or thrice with a 24 days interval between each addition. B) At 6 months Anti-TGF was added to the system, using an event. Anti-TGF was added three times to the model with either a 1-day, 6-day, 12-day or 24-day interval between each addition. C) At 15 months Anti-TGF was added to the system, using an event. Anti-TGF was added either once, twice or thrice with a 24 day interval between each addition. D) At 19 months Anti-TGF was added to the system, using an event. Anti-TGF was added three times to the model with either a 1-day, 6-day, 12-day or 24-day interval between each addition.

1D11 is an antibody that targets TGFβ, leading to its degradation. It has been demonstrated that injection of this antibody into the subchondral bone can attenuate surgically induced OA progression in mice and rats [33], and so has promising clinical applications for human OA. We modelled the addition of the antibody by adding a species (Anti-TGF) which could bind to TGFβ and degrade it in both its inactive and active forms. We also assumed that Anti-TGF degrades over time and leave the system. It took Anti-TGF about 34 hours to completely leave the system, the same length of time as was reported in a murine model of OA [34] (S8 Fig). Simulated injections of Anti-TGF were modelled at different time-points and the effect on *MMP13* mRNA was examined over a 30 month time period (Fig 6). A single “injection” at 6 months, led to an increase in *MMP13* mRNA. A second injection, 24 days later helped to reduce the levels of *MMP13* mRNA at later time-points. However, a third injection after another 24 days dramatically reduced the levels of *MMP13* mRNA. (Fig 6A). Having discovered that three “injections” reduced levels of *MMP13* mRNA, we used the model to explore different time lags between each injection (Fig 6B). The results showed that the optimal strategy was to space the simulated “injections” every 24 days.

We also examined the effect of late interventions with a single “injection” at 19 months at which time Alk1 is the dominant receptor, and TGFβ signalling has mainly detrimental effects (Fig 6C). As for the 6-month intervention, this led to an increase in *MMP13* mRNA but prevented the continual increase in *MMP13* mRNA after 25 months. A second injection 24 days later greatly reduced levels of *MM13* and a third injection after another 24 days, completely reduced *MMP13* levels and even after a pro-inflammatory stimulus at 24 months, the increase in *MMP13* was only very transitory. As for the early intervention, spacing “injections” every 24 days was more effective than short time intervals (Fig 6D) but in all cases interventions at 19 months were much more effective than the 6 month interventions (compare Fig 6B and 6D). Therefore the model predicts that later interventions when TGFβ signalling is predominantly via the Alk1 pathway, are more effective.

#### Anti-Alk1 treatment

**Fig 7:**
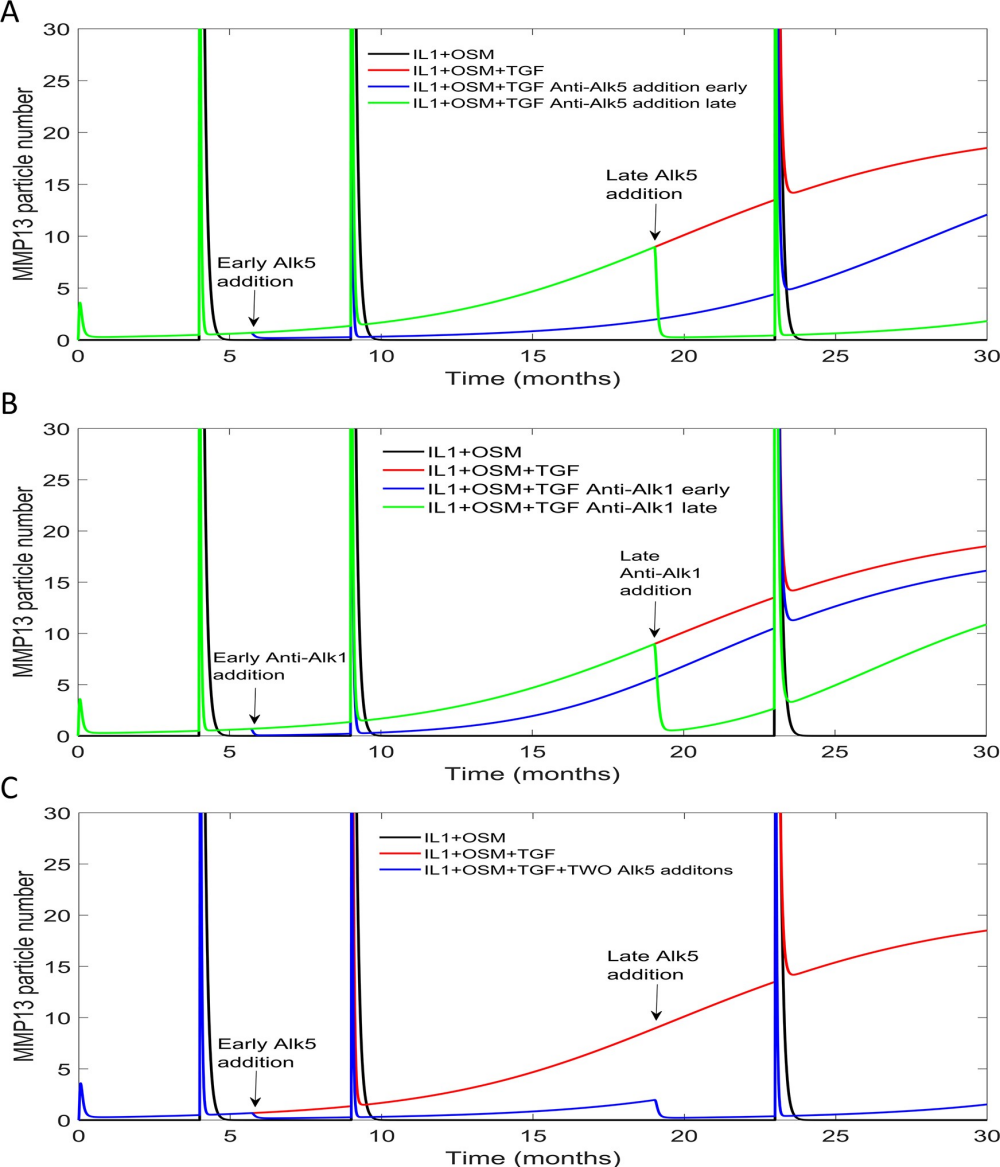
The effects of targeting type one receptors, at 6 or 19 months, on MMP-13 mRNA expression. Deterministic simulation results for the complete model, with an extra reaction to allow RUNX2 inactivation. The data show the change in MMP-13 mRNA across a 30-month simulation time. IL-1+OSM were triggered using events at 4, 9 and 23 months. A) The model also contained the species “Anti-ALK1” which targeted the ALK1 receptor for degradation. Anti-ALK1 concentration was at zero when the simulation started but at either 6 months (early) or 19 months (late) Anti-ALK1 was added to the system, using an event. B) ALK5 level were restored to its initial level of 500, using an event at either 6 months (early) or 19 months (late). C) ALK5 level were restored to its initial level of 500, using an event at both 6 months (early) and 19 months (late).

As TGF-β signalling via Alk1 is detrimental to cartilage, we also considered the effect of inhibiting Alk1 on MMP13 expression by including a species named “Anti-Alk1”. We assumed that Anti-Alk1 bound to Alk1 to promote its degradation. We simulated the addition of Anti-Alk1 at two different time points, 6 and 19 months (Fig 7A). The earlier treatment resulted in reduced *MMP13* mRNA even at later time points but there was still a steady increase over time. On the other hand, the late treatment resulted in a larger reduction of *MMP13* mRNA after 19 months, but the effect was only temporary, as although the level of *MMP13* was lower it continued to increase at later time points. These results suggest that the timing of this intervention is important. This is probably due to the fact that at early time points, the Alk1/Alk5 ratio is low and so TGF-β preferentially signals through the Alk5 pathway. Our model predicts that anti-Alk1 treatment reduces MMP13 temporarily and so may delay the onset of OA suggesting that multiple interventions may be necessary when the Alk1/Alk5 ratio is high.

#### Pro-Alk5 treatment

Since TGFβ is protective when it signals through the Alk5 pathway, the model was used to test the effects of overexpressing of Alk5 (Fig 7B). Increased expression of Alk5 at an early time point (6 months) led to a reduction in the level of *MMP13* mRNA at the later time points. Increasing Alk5 levels at a later time point (19 months) led to a greater reduction in *MMP13* mRNA at >19 months, when compared to the early treatment. When both treatments were combined there was an additive effect such that *MMP13* mRNA levels remained at low levels and the TGFβ mediated repression of the pro-inflammatory stimulus was still seen at late time points when the Alk1/Alk5 ratio would normally be high (Fig 7C).

### Bioinformatics analysis confirms the importance of inflammation in OA

**Fig 8:**
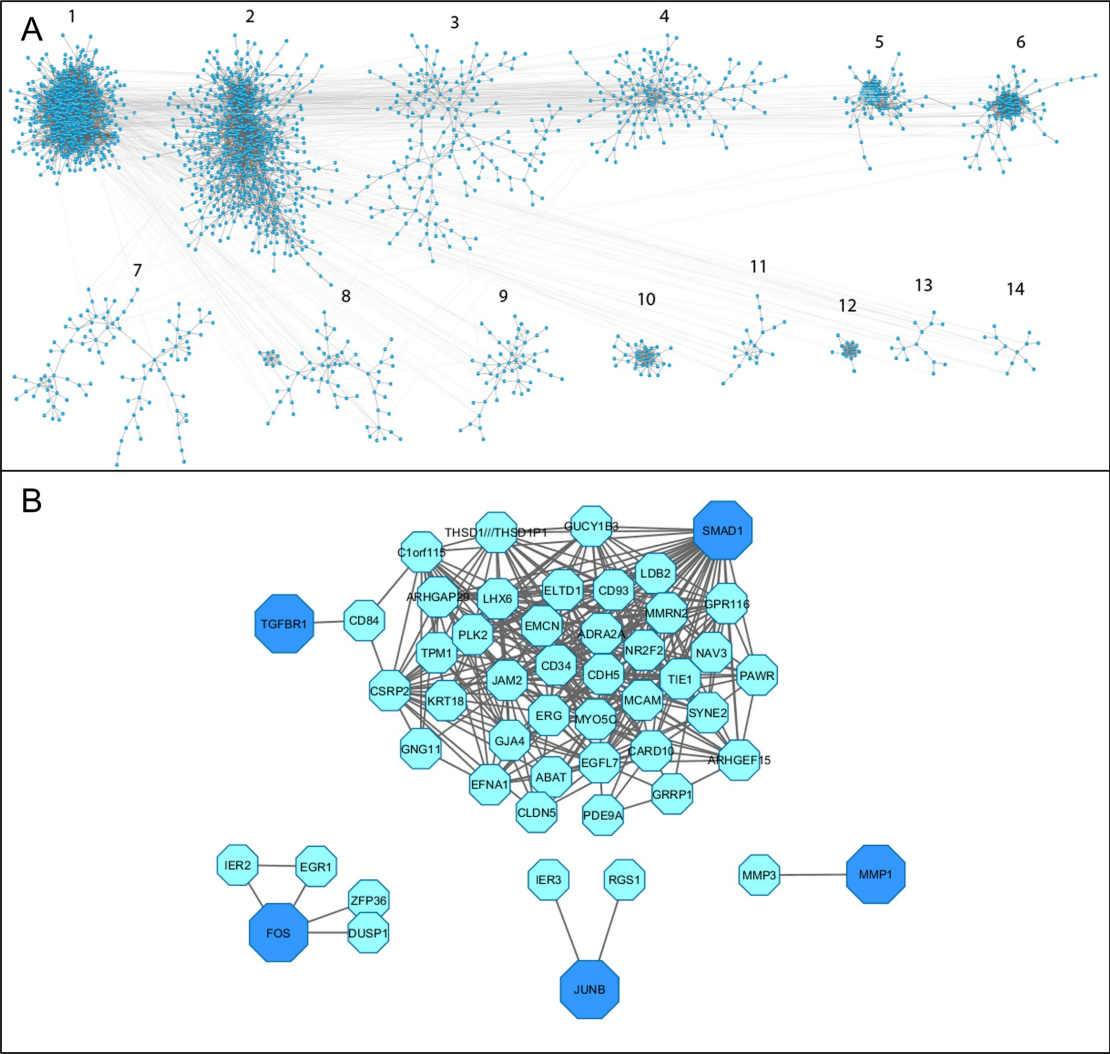
Top OA cytoscape clusters identify key model components. (A) Affymetrix microarray data from 50 knee OA patients were collected from the Gene Expression Omnibus database, normalised, and the differentially expressed genes combined. ARACNE then inferred the interactions between these genes providing mutual information (MI) values that could be visualised using Cytoscape. The nodes (blue dots) represent differentially expressed genes and the edges are the MI values. Using clusterMaker these genes were then clustered based on MI values. The 14 largest clusters are represented here as they showed significant enrichment during DAVID analysis. (B) Overlaying the model genes to this clustered network highlighted the genes that were present. Genes shown to be present (dark blue) are *SMAD1*, *TGFBR1* (Alk5), *FOS* (cFos) and *JUNB*. These were extracted along with all the genes that directly interacted with them (blue), to create the sub-networks shown.

We used publically available microarray data to examine the overall network of genes involved in human OA and to identify interactions between the IL-1, OSM and TGFβ pathways. DAVID analysis of the clustered Cytoscape network we created to represent human OA (Fig 8A) highlighted their function. Only the top 14 clusters had significant enrichment, and of these clusters 1 and 5 (Fig 8A) had multiple significantly upregulated functional terms relating to inflammation (see S3 File). Tight clustering was observed for both clusters suggesting there was considerable cross-talk/interaction between the genes in each cluster. Overlaying the genes present in the model identified three specific clusters (Fig 8B). Both *TGFBR1* and *SMAD1* were located in the largest cluster, whilst both c-Fos (*FOS*) and JunB (*JUNB*) also had links to genes within the network (Fig 8B). The implications of these findings are discussed below.

## Discussion

Matrix-degrading enzymes play important roles in the development of OA, with MMP-13 considered the key collagenase [35,36]. We initially confirmed previous studies demonstrating significant repression of IL-1+OSM-induced *MMP13* mRNA in chondrocytes by TGFβ [10–12], and our findings highlighted that TGFβ needed to be present for at least 12 h for repression to occur. This repression became more pronounced with prolonged stimulation and we also found pre-treatment with TGFβ (for 6 h) could still result in significant *MMP13* repression even when removed prior to cytokine stimulation (S6 Fig). Together, these findings indicated that *de novo* synthesis of a tertiary mediator was required to effect the observed repression.

The mechanism by which TGFβ protects against *MMP13* upregulation had previously not been explored. By using siRNA silencing of *TGFBR1*, we showed that Alk5-dependent Smad2/3 signalling was required to mediate this effect. Two potential mechanisms are that this pathway leads to upregulation of components that either inhibit transcription of *MMP13* or increase the degradation of *MMP13* mRNA. Computational modelling indicated that both AP-1 complex inhibition and increased instability of *MMP13* mRNA were required to accurately match experimental data although the necessary downstream proteins have not yet been clearly identified. Despite the alterations made to the model it still matched the simulation output of the key proteins from the original models (S9–S11 Figs). In addition, AP-1 component expression was in line with data published previously by our group [17,37]. The computational model gave further insights into how TGFβ protects against inflammatory effects via the Alk5/Smad2/3 signalling pathway and demonstrated why the age-related increase in the Alk1/Alk5 ratio may be so damaging. The model also predicts that TGFβ will decrease the variability in the cellular response to an inflammatory insult, a finding that will be interesting to test in future studies.

There are a number of potential candidates for the TGFβ-mediated repression of *MMP13*. The primary candidate for AP-1 competitive inhibition is JunB as there is a substantial body of evidence showing that it is a direct downstream target of Smad3 [38,39] and works to antagonise rapid gene transcription [40–42]. Studies have shown it can displace cFos in the AP-1 complex and bind cJun [39,41]. It has also been demonstrated to limit the effects of IL-1, in particular MMP synthesis [38]. Finally, it has been shown to repress gene expression in epithelial-mesenchymal transition in cancer due to upregulation by TGFβ, demonstrating TGFβ can produce significantly high JunB expression to limit gene transcription [38]. There is also evidence that Smad2/3 can interact directly to antagonise AP-1 complex binding, in particular to cFos and cJun [43]. JunD is also a promising candidate along with the tissue inhibitors of metalloproteinases which are all strongly induced by TGFβ [44].

How TGFβ leads to *MMP13* mRNA degradation is not currently known but there are a number of potential mechanisms. Both vinculin and far upstream element (FUSE) have been shown to bind to the 3’ UTR of *MMP13* which in turn promotes the decay of *MMP13* mRNA [45]. Sirtuin 2 (Sirt2) has also been shown to supress *MMP13*, possibly as a result of TGFβ [46]. Reports have also suggested that histone deacetylase (Hdac)4 can repress *MMP13* [47] by binding to the *MMP13* promotor [48] preventing further transcription. This protective effect has been questioned by other reports claiming the opposite is true with Hdac4 responsible for an increase in *MMP13* [49,50]. Lastly, TGFβ leads to upregulation of a number of microRNAs [51] which are known to mediate their effect by increasing mRNA degradation. For example, miR-27a is upregulated by TGFβ [52] and has been shown to target *MMP13* mRNA [53]. Determining how TGFβ mediates both AP-1 complex inhibition and mRNA degradation may identify specific therapeutic targets to help reduce cartilage damage with age and OA progression. The addition of active TGFβ to cultured medium has been widely used for engineered cartilage growth but it has been shown that there is a gradient of TGFβ in tissue constructs due to its high rate of depletion via cellular internalisation [54]. Although the turnover of TGFβ was not included in the current model, it would be straightforward to extend the model to include additional reactions. This would be an important modification if the model was to be used to examine TGFβ signalling in the context of tissue engineering.

TGFβ signalling shifts towards Alk1 responses with age [21,23]. Our model predicted that despite Alk1 signalling leading to an increase in *MMP13* [21] and cFos phosphorylation [55], there is still some degree of repression to the IL-1+OSM-mediated *MMP13* upregulation, although greatly reduced. This may be because Alk1 stimulates *MMP13* upregulation through a different mechanism to IL-1+OSM. Alk1 is believed to upregulate *MMP13* via Smad1 which can activate Runt related transcription factor 2 (Runx2) and lead to increased *MMP13* [56]. Conversely, IL-1+OSM induces *MMP13* via AP-1 components [17]. As a result, the remaining Alk5 dimers can still provide some repression as Alk1 has little direct effect. Despite this, when Alk1 was the dominant receptor the increase in IL-1+OSM-induced *MMP13* mRNA took longer to return to basal expression levels (compared to when Alk5 was dominant). As the basal expression was already high, this would lead to a longer and more pronounced response that could result in damage to already compromised tissue.

TGFβ is stored in a latent form in the synovial fluid [57] and is constantly being activated in the joint [58]. This release and activation is increased when under load or during exposure to inflammation [59,60]. There are no reports of this changing with age, but if TGFβ is dysregulated in older organisms, the release of TGFβ in response to inflammation could result in increased damage compared to younger counterparts, as a previously protective pathway changes to a catabolic pathway. Identifying how the TGFβ response has changed with age may provide therapeutic targets to help limit such damage in aged individuals, averting cartilage damage and OA development or progression.

We used the model to test the effect of potential therapeutic treatments by simulating interventions that would ameliorate the damaging effects of TGFβ. The model predicted that simulated treatments with anti-TGF, anti-Alk1, or Alk5 addition were all more effective if given at later time points when Alk1 is the predominant receptor, i.e. the timing of interventions may depend on the Alk1/Alk5 ratio. These predictions could be tested in a cellular model of OA in future work. However, we are currently unaware of any antibodies for Alk1 but hopefully they will be available in the future so that these model predictions could be tested. Although overexpression of Alk5 has not yet been tested as a potential treatment for patients, the model suggests that this may be a particularly effective treatment as it prevents Alk1 from becoming the dominant receptor. Testing the predictions in a future study will give further insights into the validity of the assumptions and may suggest further model refinements. Modelling and experimental work is an iterative process. Although we reach points where new insights have been gained, and the model can be published and made publicly available, the cycle may continue with models being re-used, adapted and tested by new experimental data [61].

Although we primarily used deterministic simulations for the computational modelling, stochastic effects can be important in biological systems such as variability in cellular responses. Stochastic modelling predicted that TGFβ reduced the variability of a pro-inflammatory response and when the system was pre-treated with TGFβ this reduced variability was even more pronounced (S6B Fig). Although it can have negative effects in disease, inflammation is an important biological response that helps to eliminate the consequences of cell injury, degrade necrotic cells and the damaged parts of tissues, as well as initiating repair [62]. Therefore, inflammatory responses are normally beneficial and it is only when they become chronically activated that damage and disease progression occur [63]; it is therefore important that they are tightly regulated. The variability of the IL-1+OSM response could lead to damage, with high levels of MMP-13 leading to cartilage degradation [64]. Conversely, insufficient upregulation of *MMP13* could result in defective tissue turnover [65]. Thus, TGFβ may help regulate inflammatory responses by reducing variability which may be lost due to ageing and promote the development and/or progression of OA.

**Fig 9:**
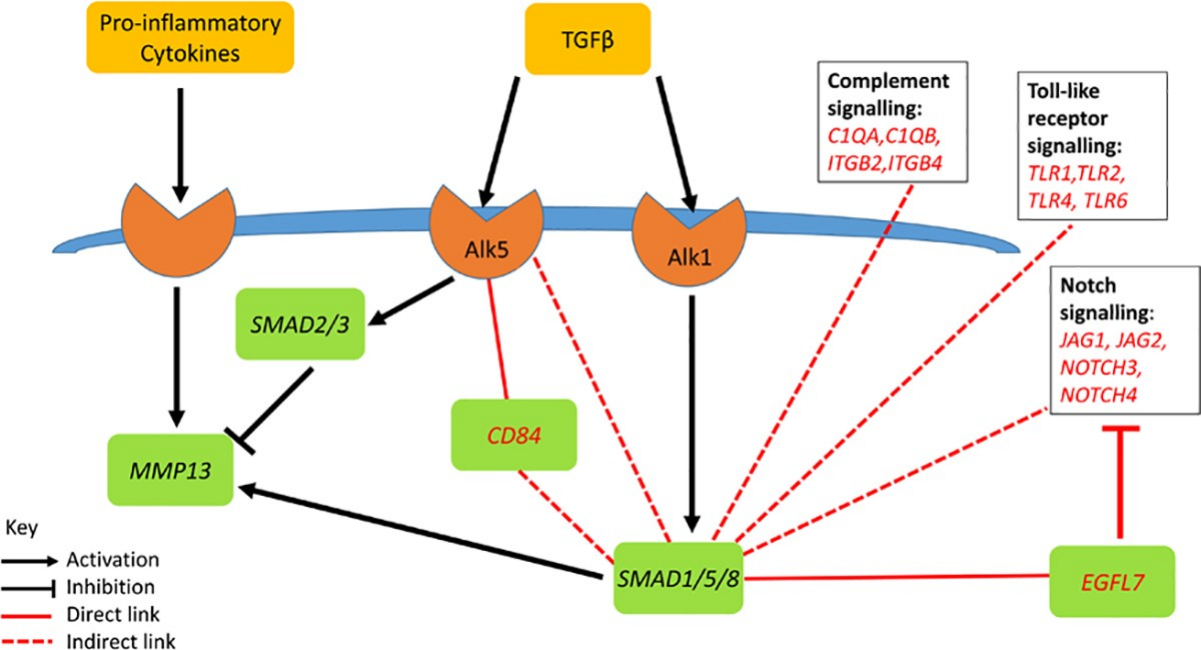
Possible extensions to the model suggested by the Cytoscape network. A simplified diagram of the model showing upregulation of *MMP13* by pro-inflammatory cytokines, the protective effect of the TGFβ/Alk5/Smad2/3 pathway and the detrimental effect of TGFβ/Alk1/Smad1/5/8 pathway. The Cytoscape network suggests crosstalk between *SMAD1* and other signalling pathways. Model components and interactions are shown with black font and lines respectively. Genes and interactions not currently in the model are shown in red.

Since multiple pro-inflammatory pathways have been implicated in the initiation and progression of OA, we focused our attention on the potent cytokine stimulus of IL-1+OSM which we recently demonstrated has considerable overlap with many other pro-inflammatory mediators [17]. Using publically available microarray data allowed us to create a Cytoscape network representative of human OA, with which we could contextualise our model and identify other pathways IL-1, OSM and TGFβ may interact with. Overlaying our model genes provided biological insight into how gene changes in the model can interact with a range of different pathways (Fig 9). Specific gene interactions highlight how changes in genes from one pathway can then lead to crosstalk with other pathways, altering them and helping to drive disease progression. This analysis identified four genes present in the model: *TGFBR1*, *SMAD1*, *FOS* and *JUNB*. *TGFBR1* was only directly linked to *CD84*, a membrane receptor of the signalling lymphocyte activation molecule family. Although the analysis showed that *TGFBR1* and *CD84* are significantly co-expressed, there is currently no evidence in the literature to support a functional relationship between them. Therefore this could be a novel connection warranting further investigation. *CD84* indirectly links *TGFBR1* to multiple genes, most of which are already linked to *SMAD1*. This indicates that *SMAD1* may affect multiple genes that are dysregulated in OA, and highlights the importance of catabolic TGFβ signalling. The gene with the strongest connection to *SMAD1* was EGF-like domain multiple 7 (*EGFL7*)—a gene linked to calcium iron binding, Notch signalling and OA [66]. Although not directly linked to *SMAD1*, there were multiple genes in cluster 1 related to Notch, Complement and the Toll-like receptor signalling pathways, providing further evidence of crosstalk between pathways in OA (Fig 9). *JUNB* was linked to the immediate early response 3 (*IER3*) gene, which suggests not only a link to ERK signalling but also plays a key role in cellular stresses such as apoptosis [67,68]. Regulator of G-Protein Signalling 1 (*RGS1*) was also linked to *JUNB*. *RGS1* has been linked to the mechanical loading response [69], as well as pain in OA patients [70]. cFos and early growth response 1 (EGR1) have been shown to be co-regulated [71] and this may be important in the inflammatory response and OA development [72–74]. Also closely linked to cFos was ZFP36 ring finger protein, a protein that binds to AU-rich element-containing mRNAs to promote their degradation [65]. There is a cFos, cJun and AP-1 binding site in the *ZFP36* promoter suggesting its expression may be increased in OA [75,76]. Further exploration into these interactions and incorporating them into the model could help to identify interesting targets for future study and possible drug interventions.

This study does have some limitations. Firstly, it was necessary to use a chondrosarcoma cell line (SW1353) which is not ideal as it has been shown that they have a different gene expression profile to primary human articular chondrocytes (HACs) [77]. However, HACs were not readily available and would not have provided sufficient data for the construction and testing of the computational model. SW1353 cells are a well-established model of inflammation producing a similar catabolic response similar to HACs [77,78] and so was a reasonable choice for this study. Secondly, we only examined the effect on gene expression after stimulation with TGFβ, IL1+OSM+TGFβ, or knockdown of *ALK5* in this study. It would be particularly interesting to examine the effects of knocking down *ALK5* on levels of phospho-Smads in a future study to examine whether or not the increase in *MMP13* is due to a switch from Alk5 to Alk1 signalling.

Computational models have limitations as it is necessary to make simplifying assumptions. The assumptions of the model presented here were based on current knowledge, enabling the model to capture the behaviour of the experimental system under different conditions. The model was based on two previous published models [25,79] and it was necessary to reduce the complexity of integrated model. Therefore we made further simplifying assumptions but showed that this had little effect on the model output (S9–S11 Figs). In order to combine the previous models, we needed to make further assumptions regarding the effect of TGFβ signalling on *MMP13* expression and although this allowed us to match our model to experimental data, there may alternative mechanisms that have yet to be explored.

In summary, we have shown that TGFβ can repress *MMP13* expression and reduce the variability of an inflammatory response which, together, limit joint damage. We confirm that in chondrocytes this is a result of Alk5 receptor signalling whilst computational modelling predicts that receptor changes towards an Alk1-dominant phenotype with age could result in prolonged damage from inflammation. The model output suggests that this is due to both an increase in basal levels of *MMP13* and a significant reduction in the protective effect of TGFβ after an acute pro-inflammatory event. Herein, we have highlighted the advantages of combining computational modelling, bioinformatics and experimental techniques to more comprehensively explore a system and potentially identify new therapeutic targets for further study.

## Materials and methods

### Chemicals

IL-1α was a generous gift from Dr. K. Ray (GSK, Stevenage, UK), whilst OSM was generated in-house. TGFβ was purchased from Peprotech (Rocky Hill, USA). All chemicals used for reverse transcription were purchased from Thermo Fisher (Loughborough, UK). The primers and master mix components for real-time PCR (qPCR) experiments were designed and purchased from Sigma-Aldrich (St. Louis, USA) unless stated otherwise.

### Cell culture

The human chondrosarcoma cell line, SW1353, was purchased from American Type Culture Collection (catalogue no. HTB-94; Rockville, MD). Cells were cultured at 37°C in a medium containing Dulbecco's Modified Eagle’s Medium/Nutrient Mixture F-12 (DMEM/F-12) (Sigma-Aldrich) supplemented with 1% glutamine, non-essential amino acids, penicillin (100 IU/ml) and streptomycin (100 μg/ml) plus 10% foetal calf serum (FCS). Cells were cultured in serum-free medium overnight prior to stimulation.

### Real-time PCR

RNA extraction and cDNA synthesis were performed using the Cells-to-Signal^TM^ kit (Life Technologies, Carlsbad, USA) as directed. mRNA levels of genes were obtained from standard curves and corrected using GAPDH or 18S ribosomal RNA levels following TaqMan PCR according to the manufacturer’s instructions (Applied Biosystems, Warrington, UK). Oligonucleotide primers were designed using Primer Express software version 1.0 (Applied Biosystems), to be in different exons close to, or spanning, an exon boundary so as to prevent amplification of residual cDNA. The sequences of primers and probes used were as previously described [80], or: *GAPDH*: For, 5’-GTGAACCATGAGAAGTATGACAAC-3’; Rev, 5’-CATGAGTCCTTCCACGATACC-3’; Probe, 5’-CCTCAAGATCATCAGCAATGCCTCCTG-3’; *TGFBR1*: For, 5’-GCAGACTTAGGACTGGCAGTAAG-3’; Rev, 5’-AGAACTTCAGGGGCCATGT-3’; Universal Probe Library (Roche, Burgess Hill, UK) Probe #5; *ACVRL1*: For, 5’-AGACCCCCACCATCCCTA-3’; Rev, 5’-CGCATCATCTGAGCTAGGC-3’; Probe #71; *ID1*: For, 5’-CCAGAACCGCAAGGTGAG-3’; Rev 5’-GGTCCCTGATGTAGTCGATGA-3’; Probe #39; *PAI1*: For, 5’-AAGGCACCTCTGAGAACTTCA-3’; Rev, 5’-CCCAGGACTAGGCAGGTG-3’; Probe #19. The relative gene expression in samples was then determined using either a Lightcycler (Roche Diagnostics, Indianapolis, USA) or a 7900HT PCR system (Applied Biosystems).

### Immunoblotting

Protein extraction was performed using a previously described protocol [80]. Lysates were resolved by sodium dodecyl sulfate polyacrylamide gel electrophoresis, using a 12% acrylamide gel. Proteins were then transferred to nitrocellulose membranes, and subsequently probed overnight with the following antibodies: anti-Alk5 (Abcam, Cambridge, UK) at 1:1000 and anti-GAPDH (Millipore, Watford, UK) at 1:30000.

### siRNA-mediated gene silencing

SW1353 chondrocytes were serum-starved overnight prior to treatment for 24 h with 50 nM ON-TARGET Plus siRNAs (Dharmacon, Colorado, USA) in serum-free antibiotic containing medium and Dharmafect1 transfection reagent (Dharmacon), following the manufacturer’s protocol. Cells were washed to remove siRNA and transfection reagent before cytokine stimulation.

### Model construction and simulation

An integrated model was created based upon two previously published models, one representing the pro-inflammatory stimulus IL-1+OSM [25] and another of simulated age-related changes in murine joints which contained a TGFβ component [23]. The model assumptions are given in S2 File together with full details of all components and reactions. The model was constructed in complex pathway simulator (COPASI) [81]. Deterministic simulations were run using the simulation algorithm LSODA [82]. Simulations were configured with the following parameters: Duration (1440), Interval Size (1), Relative Tolerance (1e-06), Absolute Tolerance (1e-12), Maximum Internal Steps (10000). Stochastic simulations were run using the direct method, which uses the Gillespie algorithm [83]. Simulations were configured with the maximum internal step set at 1000000 whilst random seed was not used. COPASI was also used for parameter estimations, primarily using the genetic algorithm (with the parameters: number of generations (1000), population size (20), random number generator (1) and seed (0)), with further refinements using the Hooke and Jeeves algorithm (with the parameters: tolerance limit (1e-08), iteration limit (50) and Rho (0.2)). The model was exported in Systems Biology Mark-up Language (SBML) [84], whilst simulation data were exported from COPASI and visualised using MATLAB r2013a (MathWorks Inc., Natick, USA), via the built-in graph feature. The model network diagrams were visualised using CellDesigner 4.4 [85] and Systems Biology Graphical Notation (SBGN) [86]. Simulations were run using COPASI version 4.20. The model was deposited in Biomodels [61] and assigned the identifier MODEL1805080001.

### Bioinformatics tools

To create a network overview of human OA we accessed publically available OA microarray data (all Affymetrix) from the Gene Expression Omnibus [87]. Of these, we only used diseased samples, obtaining 50 synovial samples from 3 different studies [88–90]. These were normalised and then combined using R [91]. Differences caused by experimental variation between samples were normalised using ComBat [92], followed by determination of gene interactions and the strength of these interactions using Algorithm for the Reconstruction of Accurate Cellular Networks (ARACNE) [93]. The resulting data were then imported into Cytoscape [94] and clustered to generate an overview of OA as a network. Functional analysis of the clusters was performed using the Database for Annotation, Visualization and Integrated Discovery (DAVID) [95].

## Supporting information

S1 Fig Effect of TGFβ on IL-1+OSM-induced *MMP13* expression. SW1353 chondrocytes were stimulated with IL-1 (0.5 ng/ml) and/or OSM (10 ng/ml) ± TGFβ (0.15 ng/ml or the concentration indicated) for 24 h. qPCR was then performed on isolated mRNA to measure *MMP13* expression. Data are presented as fold change relative to IL-1+OSM (mean ± SEM). Data were pooled from 5 independent experiments (n ≥ 20) (A) and from 1 experiment (n = 5–6) (B), and statistical comparisons performed using an unpaired Student’s t-test, where ***p < 0.001 vs IL-1+OSM. *GAPDH* or *18s* was used throughout for normalisation purposes. Data used to construct the figure are provided in S4 File.
(TIF)

S2 Fig Effect of *TGFBR1* silencing on TGFβ-mediated repression of *MMP13*. SW1353 chondrocytes were stimulated for 2 h with TGFβ (10ng/ml). qPCR was then performed on isolated mRNA to measure the expression of (A) *PAI1* or (B) *ID1*. Data are presented as fold change relative to control (normalized to 1.0; mean ± SEM), pooled from 3 separate experiments, n = 15–17. (C) SW1353 chondrocytes were treated for 24 h with serum-free medium or 50nM non-targeting siRNA (siCON) or a siRNA specific to *TGFBR1* (siALK5). Chondrocytes were then stimulated for 24 h with IL-1 (0.5ng/ml) + OSM (10ng/ml) ± TGFβ (10ng/ml). qPCR was then performed on isolated mRNA to measure expression of *MMP13*. Data are presented as mean fold change relative to IL-1+OSM (normalized to 1.0; mean ± SEM) with pooled data from 3 independent experiments, n = 19–20. Statistics were calculated using an unpaired Student’s t-test, where ** = p < 0.01 and *** = p < 0.001. *GAPDH* was used throughout for normalisation purposes. Data used to construct figure are provided in S4 File.
(TIF)

S3 Fig Level of *TGFBR1* mRNA relative to *ACVRL1*. SW1353 chondrocytes were serum-starved overnight and then harvested without stimulation. qPCR was then performed on the isolated mRNA to measure expression of *TGFBR1* (*ALK5*) and *ACVRL1* (*ALK1*). The data are presented relative to *ACVRL1* (normalized to 1.0; n = 10, mean ± SEM). *GAPDH* was used throughout for normalisation purposes. Statistics were calculated using an unpaired Student’s t-test, where ***p < 0.001 vs *ACVRL1*. Data used to construct the figure are provided in S4 File.
(TIF)

S4 Fig Effect of siRNA-mediated *TGFBR1* silencing on Alk5 expression. SW1353 chondrocytes were harvested following 24 h treatment with a non-targeting siRNA (siCON) or a siRNA specific to Alk5 (si*TGFBR1*), at 50 nM final concentration. (A) Whole cell lysates were resolved by SDS-PAGE and then Western blotted for Alk5 (left panel) or GAPDH (right panel). Data are representative of 3 independent experiments on separate SW1353 chondrocyte populations. (B) qPCR was performed on isolated mRNA to measure *TGFBR1* expression. Data are presented as fold change relative to siCON (normalised to 1.0; n = 5–6, mean ± SEM), using *GAPDH* for normalisation purposes. Statistics were calculated using an unpaired Student’s t-test, where ***p < 0.001 vs siCON. Data used to construct the figure are provided in S4 File.
(TIF)

S5 Fig Network diagram of the IL-1+OSM+TGFβ model. Schematic representation of the complete model detailing all species interactions between the IL-1, OSM and TGFβ signalling pathways. The blue box specifically highlights the Alk1 section of the model, whilst the red box highlights the Alk5 section.
(TIF)

S6 Fig Pre-treatment with TGFβ was sufficient to mediate repression of IL-1+OSM-induced *MMP13*. Simulation modelling of 6 h of TGFβ pre-treatment followed by an event that triggers IL-1+OSM stimulation for a further 48 h. The total pool of TGFβ was active at the start of the simulations. Curves show how the particle numbers of *MMP13* mRNA change with time. (A) Deterministic simulation results where the black arrows show the percentage repression seen at 12, 24 and 48 h, due to the presence of TGFβ. (B) Simulation results showing the average behaviour ± the standard deviations of 100 stochastic runs. The coloured shading shows the variation at each time point. (C) Pooled data from SW1353 chondrocytes treated with serum-free medium ± TGFβ (10 ng/ml) for 6 h, washed and then stimulated with IL-1 (0.5 ng/ml) + OSM (10 ng/ml) ± TGFβ (10 ng/ml) for 6–48 h. qPCR was then performed on the isolated mRNA to measure *MMP13* expression. Data are presented as fold change relative to IL-1+OSM (normalised to 1.0 at each time point; mean ± SEM). The percentages indicate the extent of repression relative to IL-1+OSM at the relevant time point. Data were from 3 separate cell populations (n = 14–19). Statistics calculated using unpaired student t-test, where *p < 0.05; **p < 0.01; ***p < 0.001. Model parameters for (A-B) are provided in S2 and S4 Files, data used to construct panel (C) are provided in S4 File.
(TIF)

S7 Fig Effects of an additional RUNX2 inactivation reaction on the model. Deterministic simulation results for the complete model, showing the change in MMP-13 mRNA across 20 months simulation time. IL-1+OSM were triggered using events at 4, 9 and 23 months. An extra reaction is added to the model, which allows RUNX2 to move from its active form to its inactive form without SMAD2 involvement. The simulations were run using COPASI. Model details are provided in S4 File.
(TIF)

S8 Fig Degradation of Anti-TGF. Deterministic simulation results showing the degradation of Anti-TGF across 50 hours simulation time. The simulation was run using COPASI. Model details are provided in S4 File.
(TIF)

S9 Fig Comparative modelling of AP-1 component profiles following IL-1+OSM stimulation. Simulation results showing the effect of IL-1+OSM treatment on the profile of the AP-1 components cFos and cJun, using a simulated time period of 5 h. Both the original IL-1+OSM model (red) described in Proctor et al. (2014) [25] and the new integrated model presented herein (blue), were simulated deterministically using COPASI. Curves show the level of (A) cFos phosphorylation; (B) cJun phosphorylation; (C) cFos/cJun heterodimer formation; (D) cJun homodimer formation. Model parameters are provided in S2 and S4 Files.
(TIF)

S10 Fig Comparative modelling of cFos/cJun heterodimer formation during a 48 hour simulation. Simulation results showing the effect of IL-1+OSM treatment on the formation of cFos/cJun heterodimers, using a simulated time period of 48 h. Both the original IL-1+OSM model (red) described in Proctor et al. (2014) [25] and the new integrated model presented herein (blue) were simulated deterministically using COPASI. Model parameters are provided in S2 and S4 Files.
(TIF)

S11 Fig Comparative modelling of SMAD signalling and TGFβ receptor expression in response to TGFβ treatment in the absence of a pro-inflammatory response. Simulation results showing the effect of TGFβ on the profiles of SMAD signalling and TGFβ receptor expression. Using a simulated time period of 20 months, both the original TGFβ model component (red) presented in Hui et al. (2016) [23] and the new integrated model presented herein (blue) were simulated deterministically using COPASI. Curves show the amount, in particle numbers, of: (A) Alk5 homodimers; (B) phosphorylated SMAD2 bound to SMAD4; (C) Alk1/Alk5 heterodimers; (D) phosphorylated SMAD1 bound to SMAD4. Model parameters are provided in S2 and S4 Files.
(TIF)

S1 File Further details relating to Figs 1–9. Means and standard deviations of all experimental data and model parameters for simulation outputs (if changed from values provided in S2 File).
(XLSX)

S2 File Model building/justification. Also includes: **Table S1 Model species.** The names, description, and initial amounts (particle numbers) of all species in the complete model. **Table S2 Model reactions.** The reactions and parameter values in the complete model.
(PDF)

S3 File DAVID functional enrichment data from the Cytoscape clusters. (XLSX)

S4 File Further details relating to S1–S11 Figs. Means and standard deviations of all experimental data and model parameters for simulation outputs (if changed from values provided in S2 File).
(XLSX)
