# Supplementary figures and images for: Systems biology reveals how altered TGFβ signalling with age reduces protection against pro-inflammatory stimuli
Source: PLoS Comput Biol. 2019 Jan 24;15(1):e1006685. doi: 10.1371/journal.pcbi.1006685 (PMC6363221; doi:10.1371/journal.pcbi.1006685)

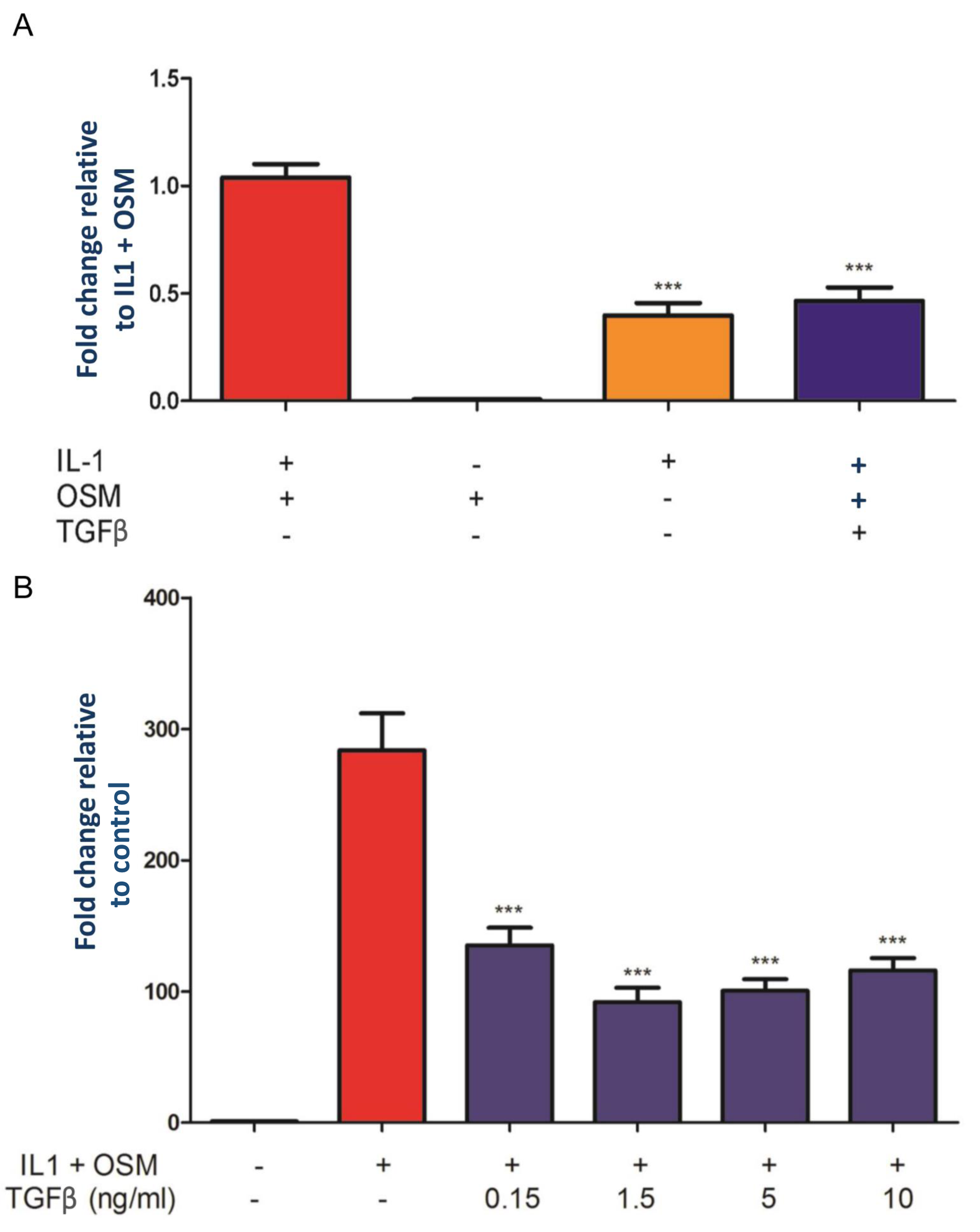

Supplement: S1 Fig — SW1353 chondrocytes were stimulated with IL-1 (0.5 ng/ml) and/or OSM (10 ng/ml) ± TGFβ (0.15 ng/ml or the concentration indicated) for 24 h. qPCR was then performed on isolated mRNA to measure MMP13 expression. Data are presented as fold change relative to IL-1+OSM (mean ± SEM). Data were pooled from 5 independent experiments (n ≥ 20) (A) and from 1 experiment (n = 5–6) (B), and statistical comparisons performed using an unpaired Student’s t-test, where ***p < 0.001 vs IL-1+OSM. GAPDH or 18s was used throughout for normalisation purposes. Data used to construct the figure are provided in S4 File. (TIF) [file pcbi.1006685.s001.tif]

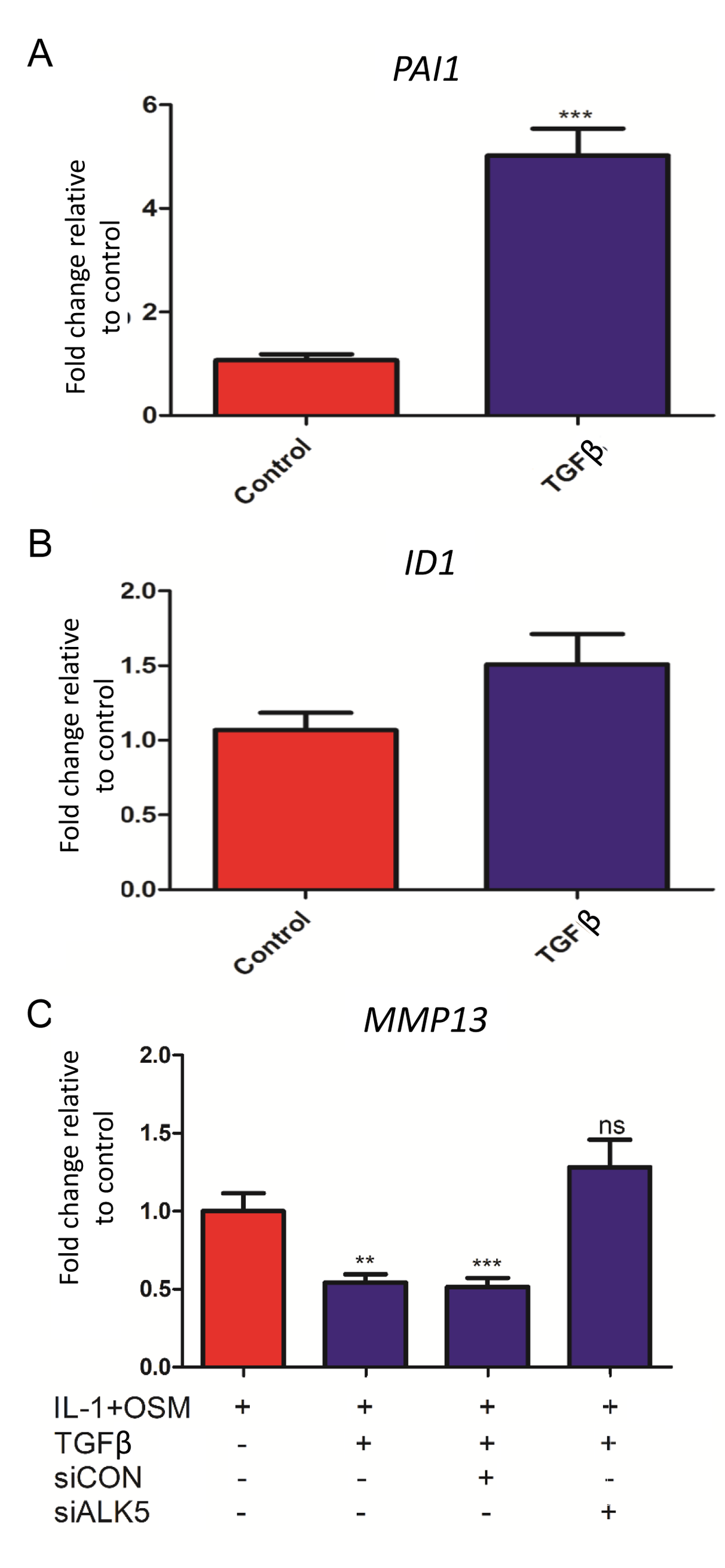

Supplement: S2 Fig — SW1353 chondrocytes were stimulated for 2 h with TGFβ (10ng/ml). qPCR was then performed on isolated mRNA to measure the expression of (A) PAI1 or (B) ID1. Data are presented as fold change relative to control (normalized to 1.0; mean ± SEM), pooled from 3 separate experiments, n = 15–17. (C) SW1353 chondrocytes were treated for 24 h with serum-free medium or 50nM non-targeting siRNA (siCON) or a siRNA specific to TGFBR1 (siALK5). Chondrocytes were then stimulated for 24 h with IL-1 (0.5ng/ml) + OSM (10ng/ml) ± TGFβ (10ng/ml). qPCR was then performed on isolated mRNA to measure expression of MMP13. Data are presented as mean fold change relative to IL-1+OSM (normalized to 1.0; mean ± SEM) with pooled data from 3 independent experiments, n = 19–20. Statistics were calculated using an unpaired Student’s t-test, where ** = p < 0.01 and *** = p < 0.001. GAPDH was used throughout for normalisation purposes. Data used to construct figure are provided in S4 File. (TIF) [file pcbi.1006685.s002.tif]

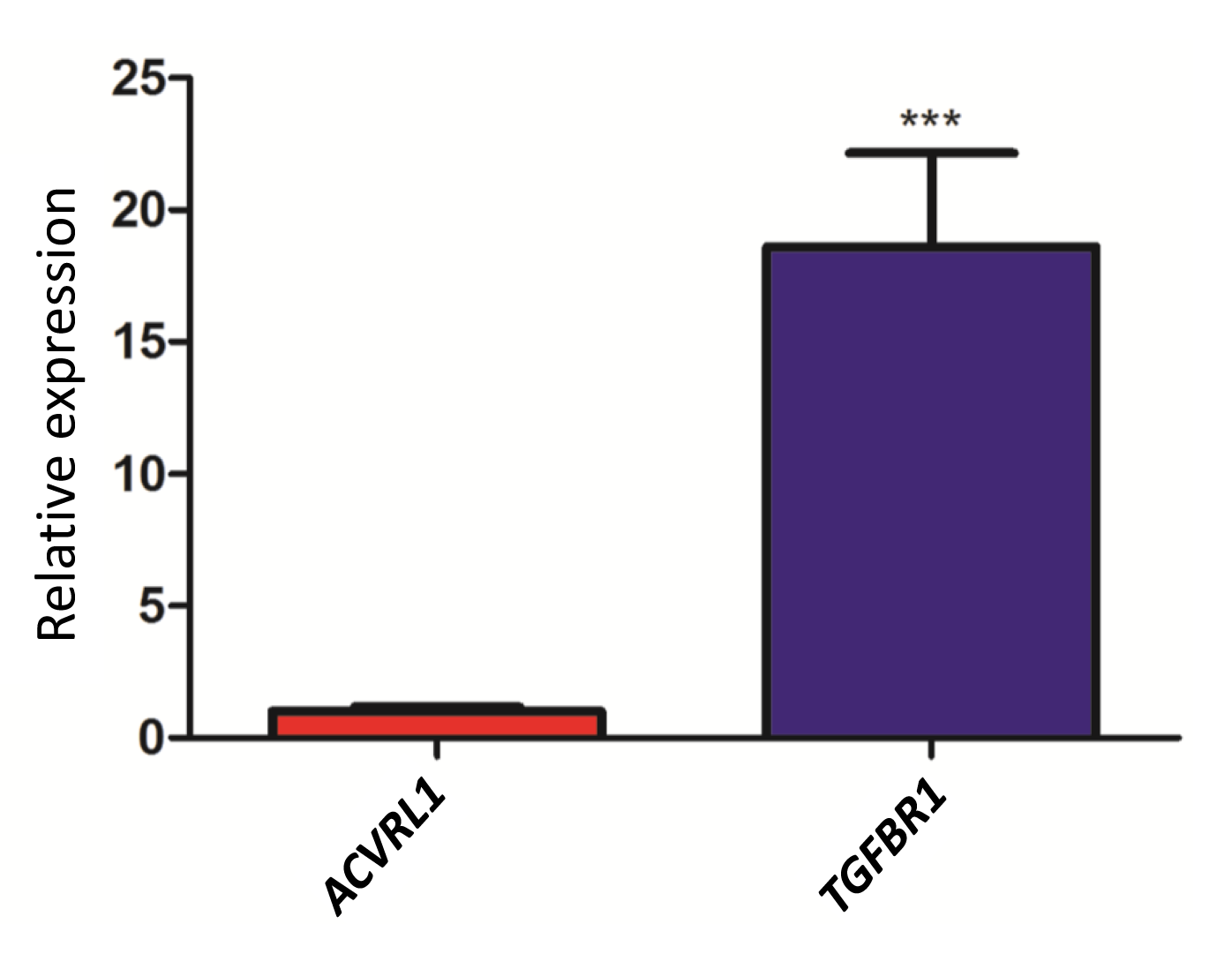

Supplement: S3 Fig — SW1353 chondrocytes were serum-starved overnight and then harvested without stimulation. qPCR was then performed on the isolated mRNA to measure expression of TGFBR1 (ALK5) and ACVRL1 (ALK1). The data are presented relative to ACVRL1 (normalized to 1.0; n = 10, mean ± SEM). GAPDH was used throughout for normalisation purposes. Statistics were calculated using an unpaired Student’s t-test, where ***p < 0.001 vs ACVRL1. Data used to construct the figure are provided in S4 File. (TIF) [file pcbi.1006685.s003.tif]

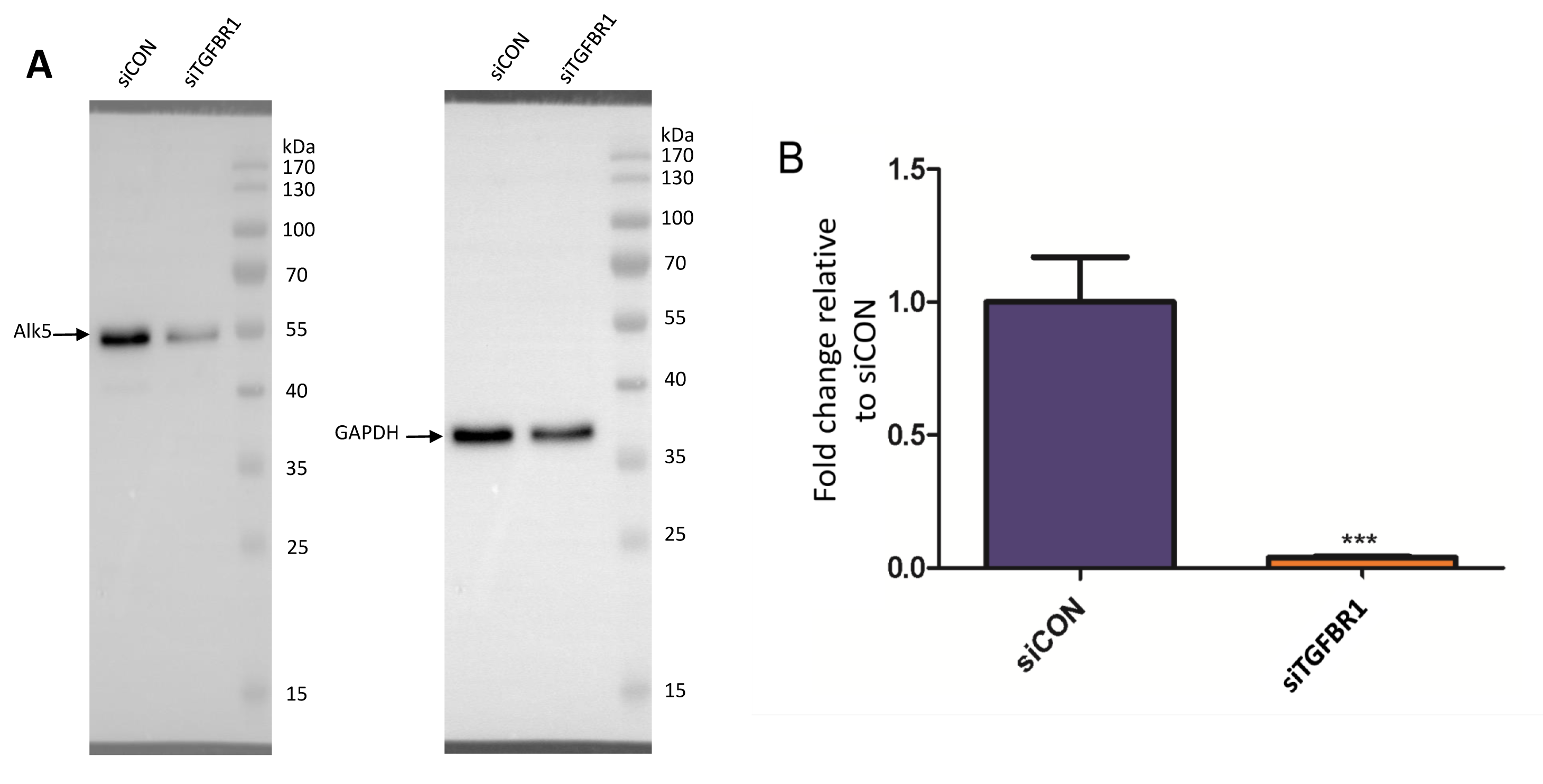

Supplement: S4 Fig — SW1353 chondrocytes were harvested following 24 h treatment with a non-targeting siRNA (siCON) or a siRNA specific to Alk5 (siTGFBR1), at 50 nM final concentration. (A) Whole cell lysates were resolved by SDS-PAGE and then Western blotted for Alk5 (left panel) or GAPDH (right panel). Data are representative of 3 independent experiments on separate SW1353 chondrocyte populations. (B) qPCR was performed on isolated mRNA to measure TGFBR1 expression. Data are presented as fold change relative to siCON (normalised to 1.0; n = 5–6, mean ± SEM), using GAPDH for normalisation purposes. Statistics were calculated using an unpaired Student’s t-test, where ***p < 0.001 vs siCON. Data used to construct the figure are provided in S4 File. (TIF) [file pcbi.1006685.s004.tif]

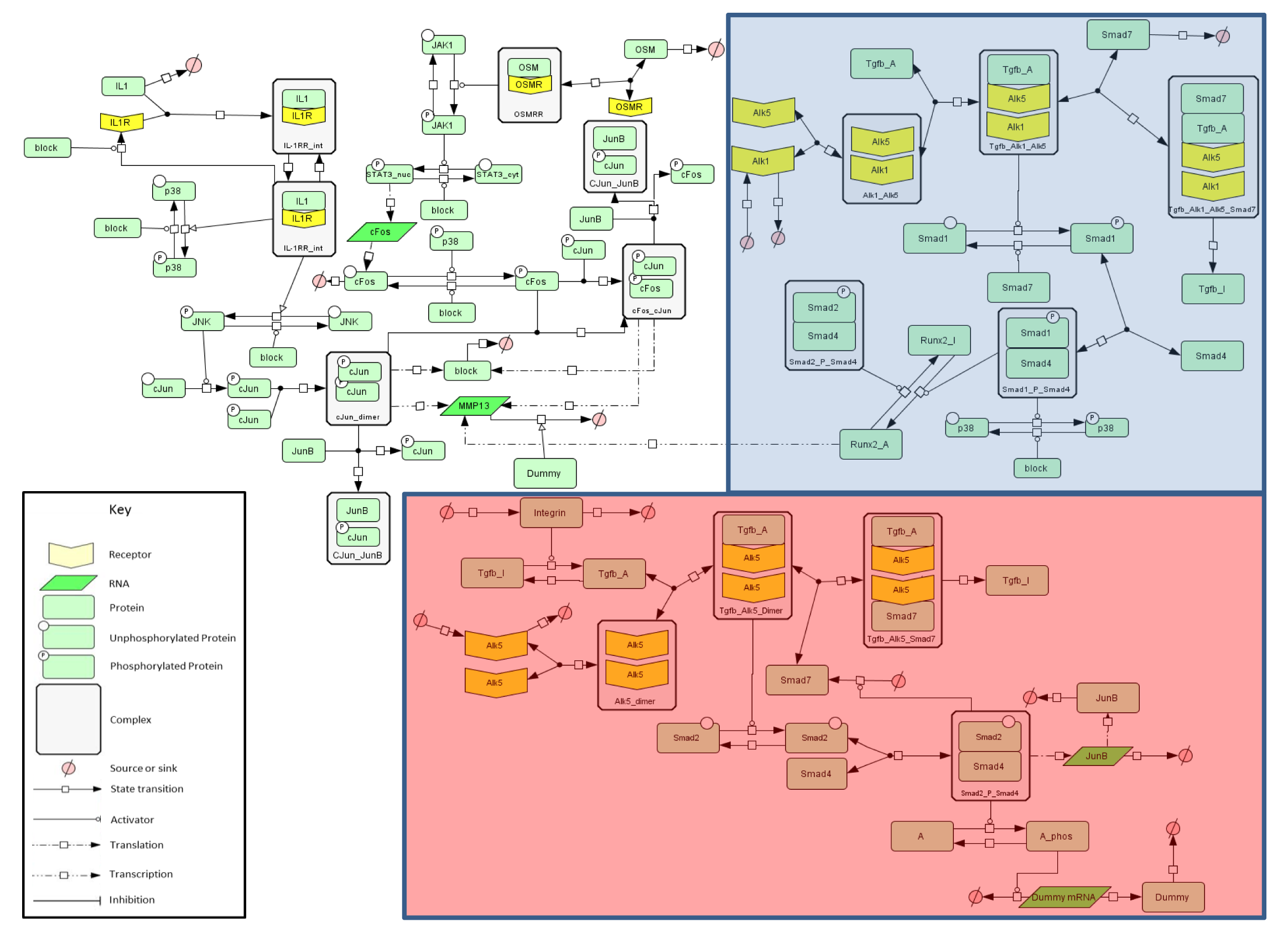

Supplement: S5 Fig — Schematic representation of the complete model detailing all species interactions between the IL-1, OSM and TGFβ signalling pathways. The blue box specifically highlights the Alk1 section of the model, whilst the red box highlights the Alk5 section. (TIF) [file pcbi.1006685.s005.tif]

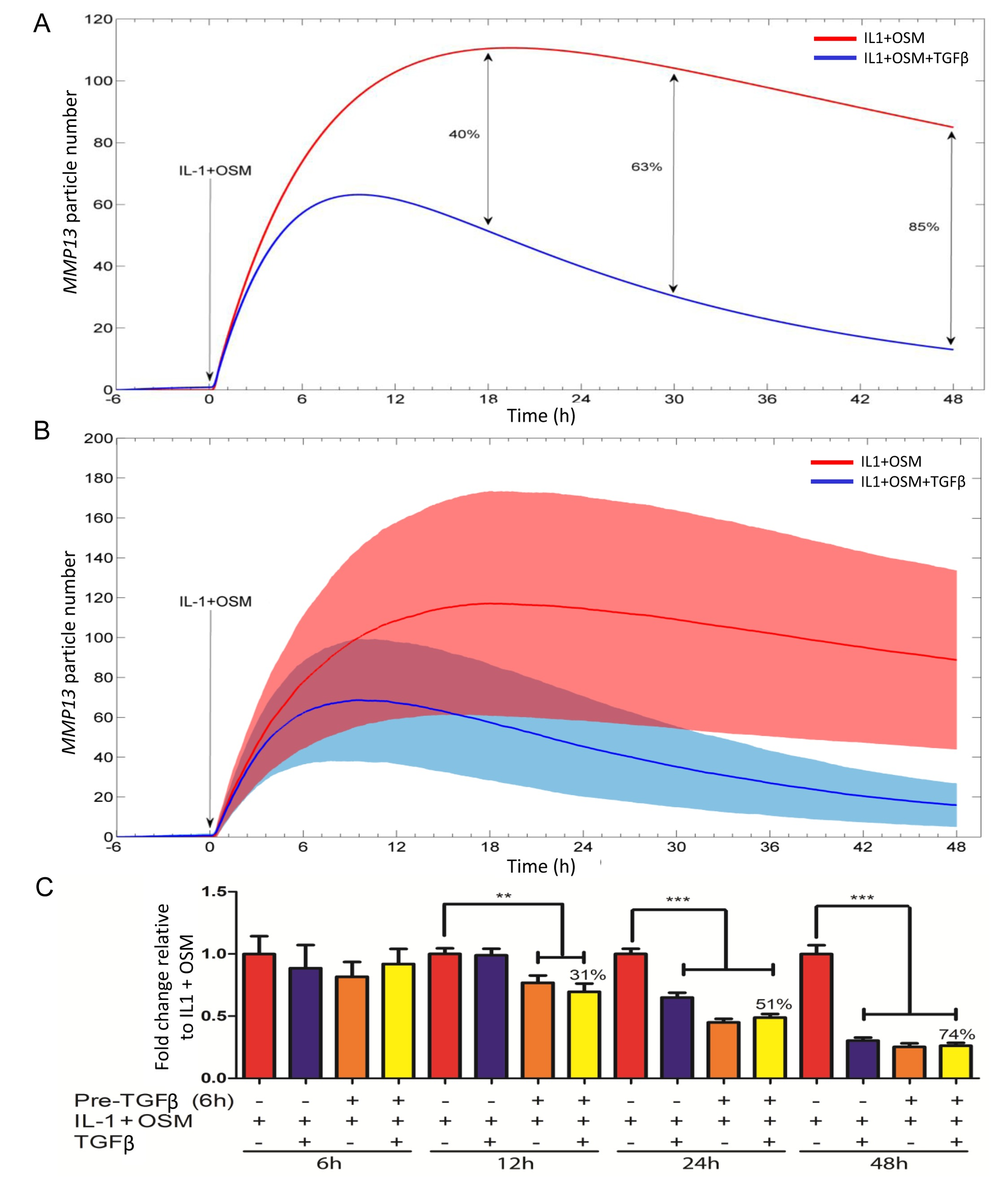

Supplement: S6 Fig — Simulation modelling of 6 h of TGFβ pre-treatment followed by an event that triggers IL-1+OSM stimulation for a further 48 h. The total pool of TGFβ was active at the start of the simulations. Curves show how the particle numbers of MMP13 mRNA change with time. (A) Deterministic simulation results where the black arrows show the percentage repression seen at 12, 24 and 48 h, due to the presence of TGFβ. (B) Simulation results showing the average behaviour ± the standard deviations of 100 stochastic runs. The coloured shading shows the variation at each time point. (C) Pooled data from SW1353 chondrocytes treated with serum-free medium ± TGFβ (10 ng/ml) for 6 h, washed and then stimulated with IL-1 (0.5 ng/ml) + OSM (10 ng/ml) ± TGFβ (10 ng/ml) for 6–48 h. qPCR was then performed on the isolated mRNA to measure MMP13 expression. Data are presented as fold change relative to IL-1+OSM (normalised to 1.0 at each time point; mean ± SEM). The percentages indicate the extent of repression relative to IL-1+OSM at the relevant time point. Data were from 3 separate cell populations (n = 14–19). Statistics calculated using unpaired student t-test, where *p < 0.05; **p < 0.01; ***p < 0.001. Model parameters for (A-B) are provided in S2 and S4 Files, data used to construct panel (C) are provided in S4 File. (TIF) [file pcbi.1006685.s006.tif]

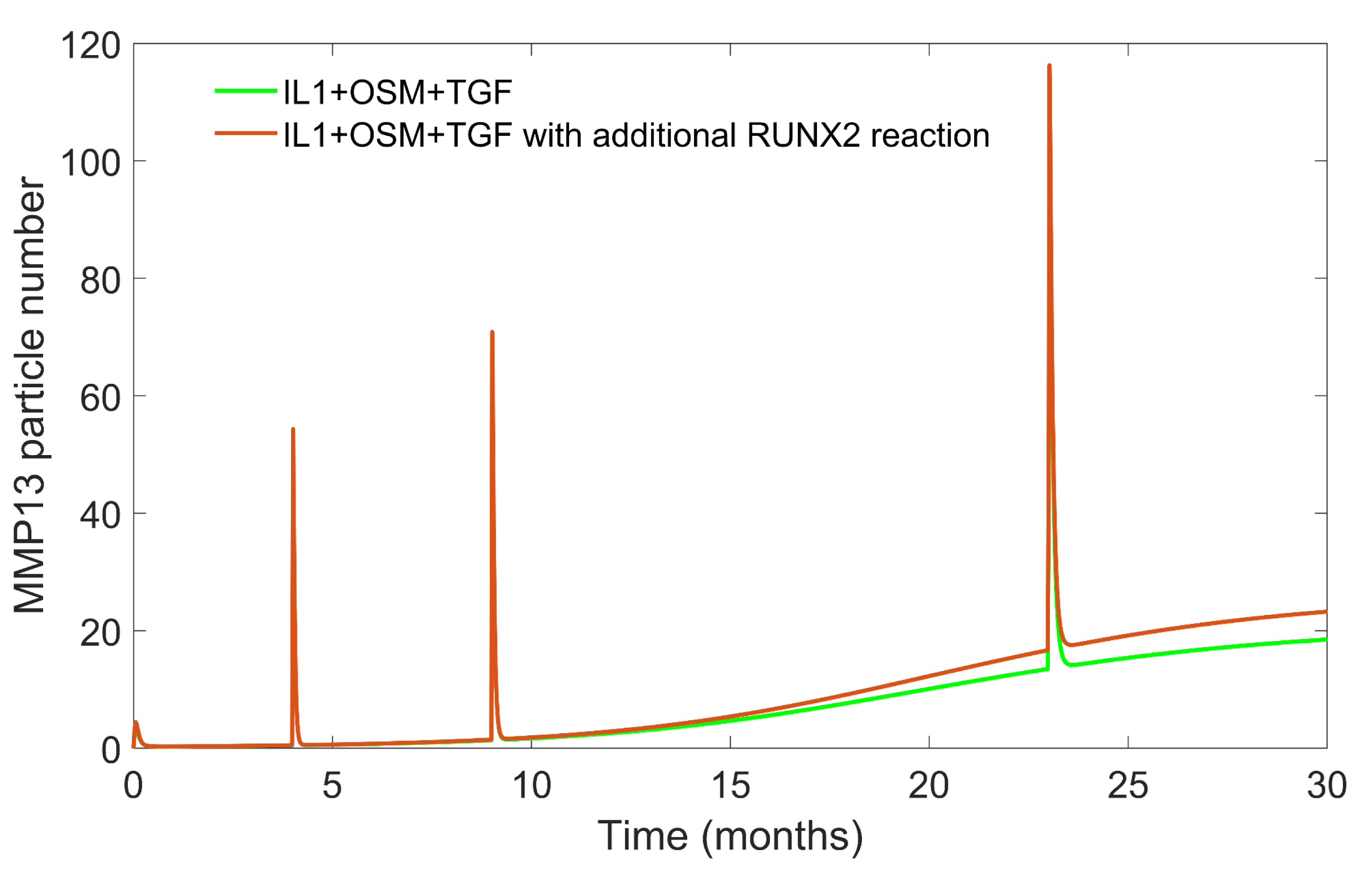

Supplement: S7 Fig — Deterministic simulation results for the complete model, showing the change in MMP-13 mRNA across 20 months simulation time. IL-1+OSM were triggered using events at 4, 9 and 23 months. An extra reaction is added to the model, which allows RUNX2 to move from its active form to its inactive form without SMAD2 involvement. The simulations were run using COPASI. Model details are provided in S4 File. (TIF) [file pcbi.1006685.s007.tif]

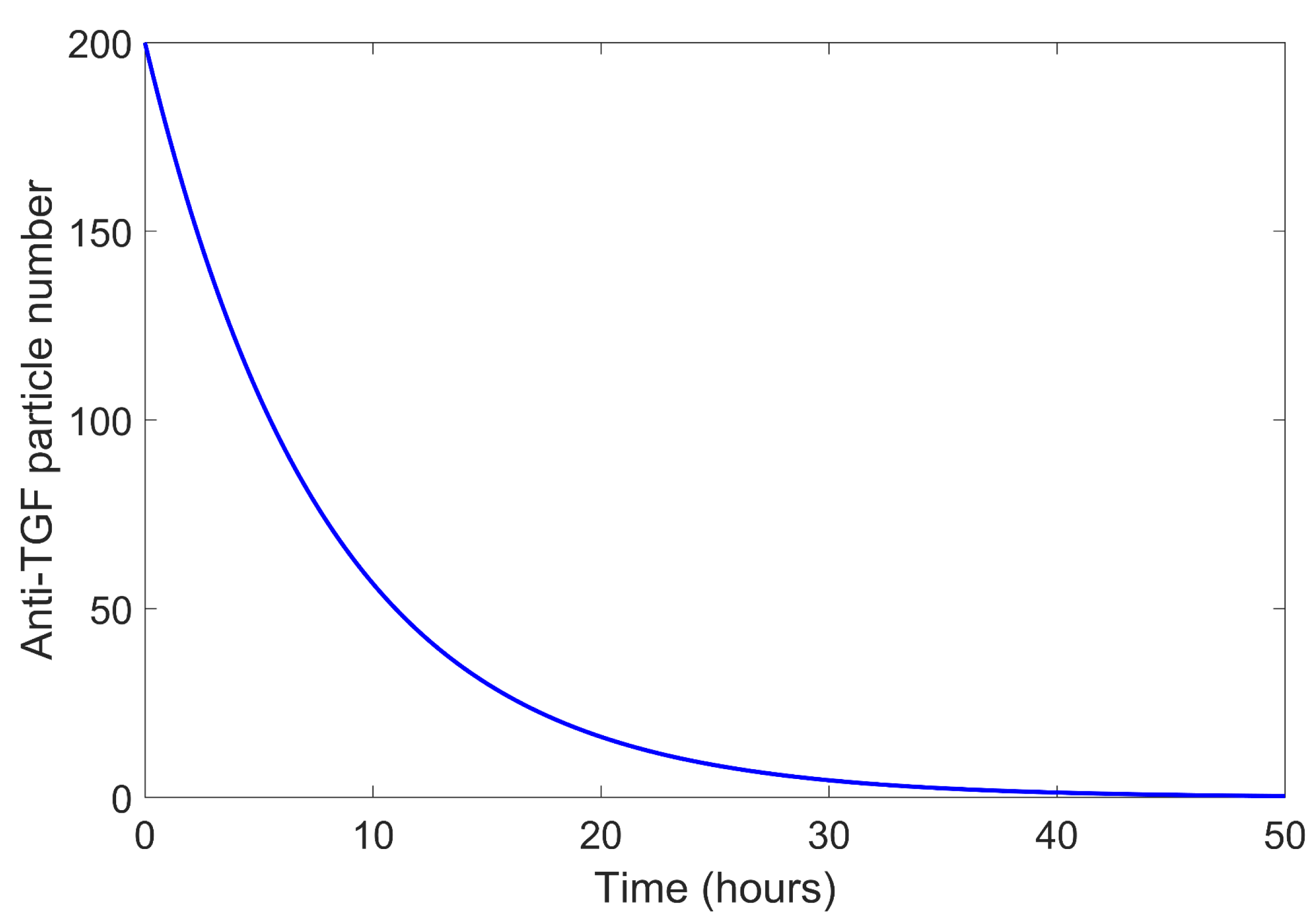

Supplement: S8 Fig — Deterministic simulation results showing the degradation of Anti-TGF across 50 hours simulation time. The simulation was run using COPASI. Model details are provided in S4 File. (TIF) [file pcbi.1006685.s008.tif]

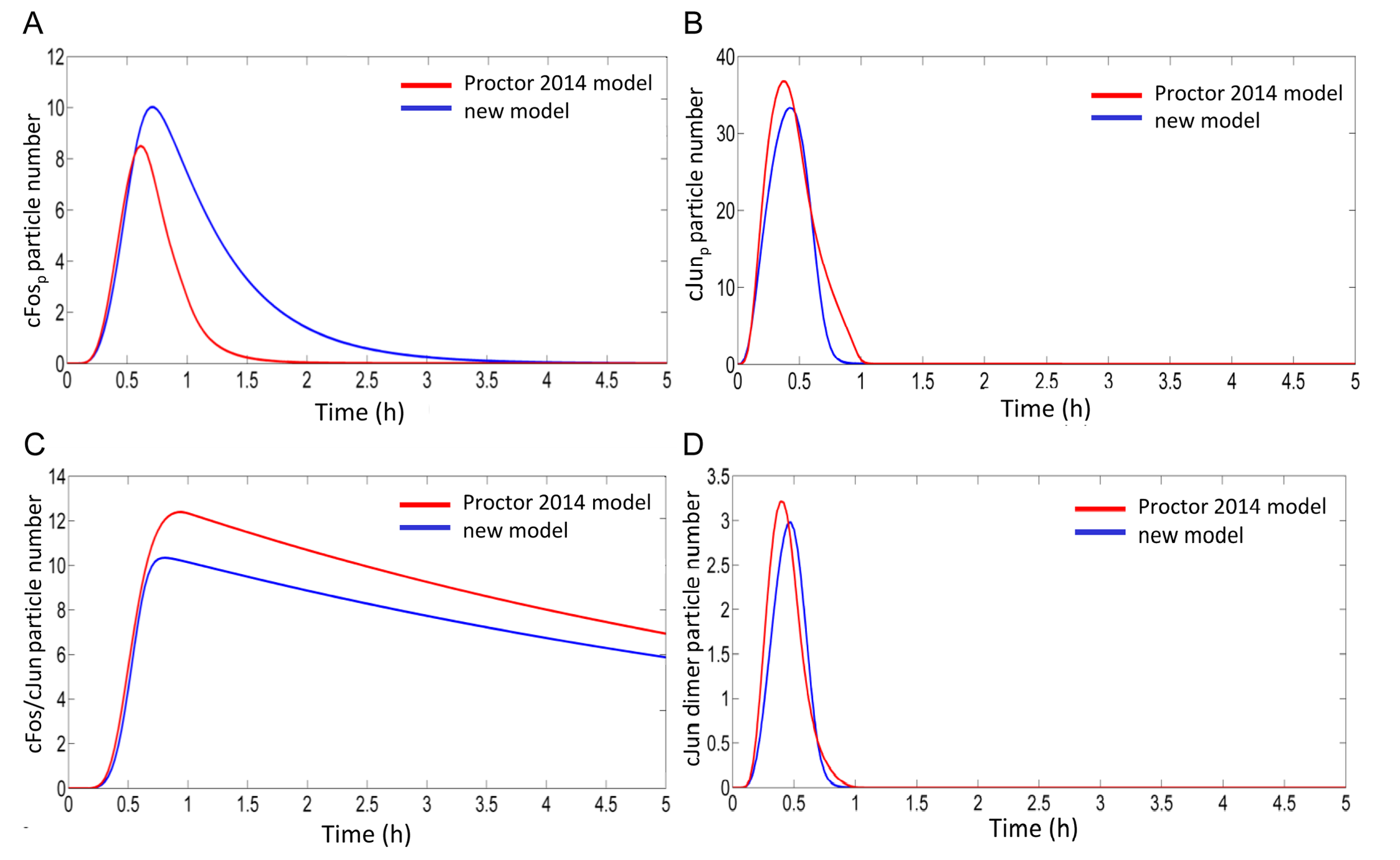

Supplement: S9 Fig — Simulation results showing the effect of IL-1+OSM treatment on the profile of the AP-1 components cFos and cJun, using a simulated time period of 5 h. Both the original IL-1+OSM model (red) described in Proctor et al. (2014) [25] and the new integrated model presented herein (blue), were simulated deterministically using COPASI. Curves show the level of (A) cFos phosphorylation; (B) cJun phosphorylation; (C) cFos/cJun heterodimer formation; (D) cJun homodimer formation. Model parameters are provided in S2 and S4 Files. (TIF) [file pcbi.1006685.s009.tif]

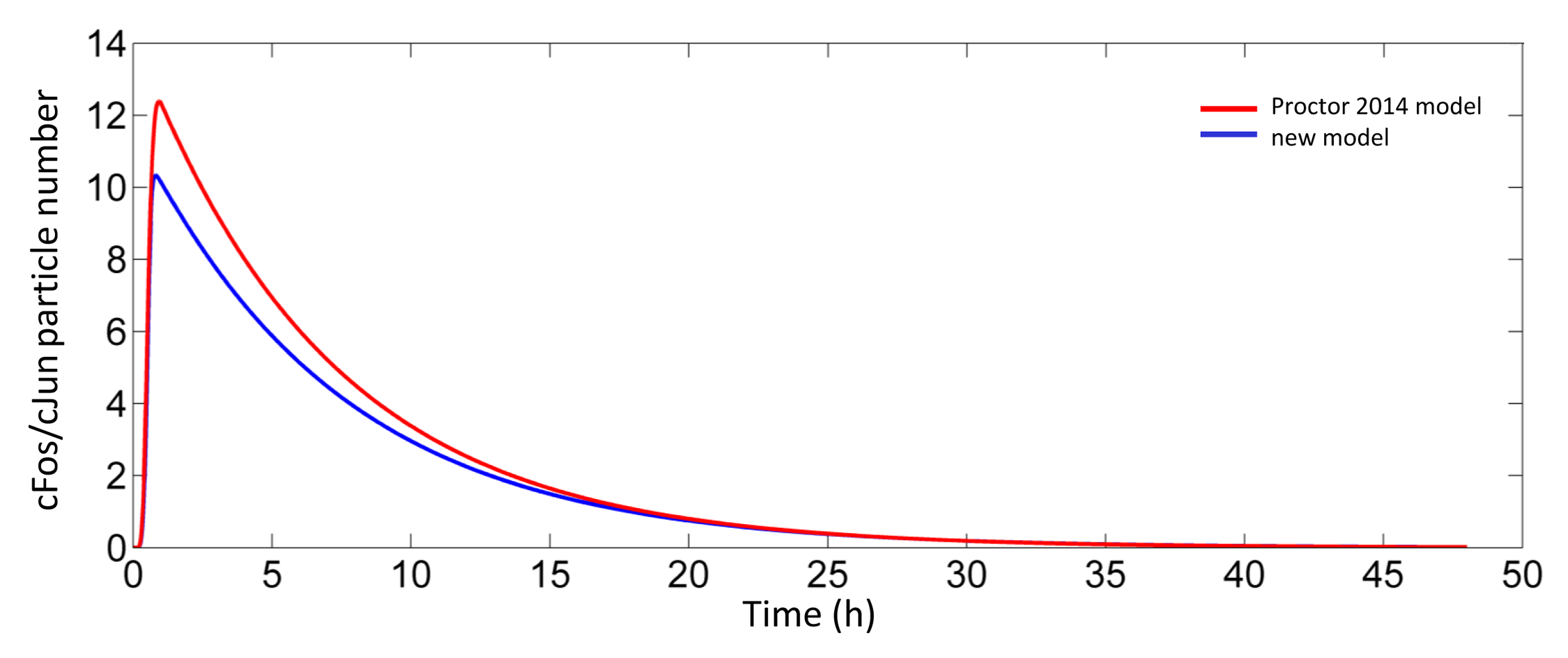

Supplement: S10 Fig — Simulation results showing the effect of IL-1+OSM treatment on the formation of cFos/cJun heterodimers, using a simulated time period of 48 h. Both the original IL-1+OSM model (red) described in Proctor et al. (2014) [25] and the new integrated model presented herein (blue) were simulated deterministically using COPASI. Model parameters are provided in S2 and S4 Files. (TIF) [file pcbi.1006685.s010.tif]

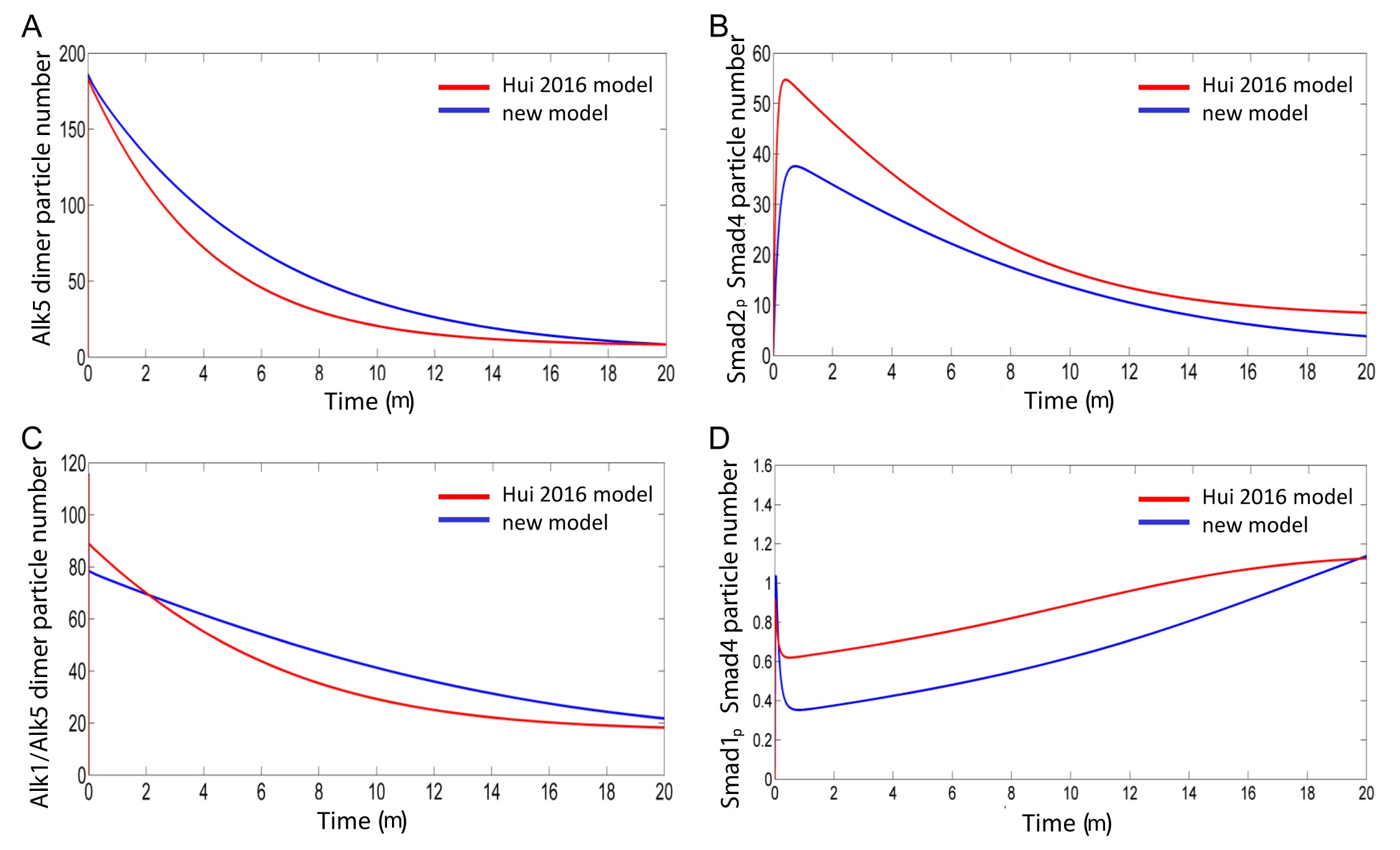

Supplement: S11 Fig — Simulation results showing the effect of TGFβ on the profiles of SMAD signalling and TGFβ receptor expression. Using a simulated time period of 20 months, both the original TGFβ model component (red) presented in Hui et al. (2016) [23] and the new integrated model presented herein (blue) were simulated deterministically using COPASI. Curves show the amount, in particle numbers, of: (A) Alk5 homodimers; (B) phosphorylated SMAD2 bound to SMAD4; (C) Alk1/Alk5 heterodimers; (D) phosphorylated SMAD1 bound to SMAD4. Model parameters are provided in S2 and S4 Files. (TIF) [file pcbi.1006685.s011.tif]
