# Supplementary material for: Systems biology reveals how altered TGFβ signalling with age reduces protection against pro-inflammatory stimuli
Source: PLoS Comput Biol. 2019 Jan 24;15(1):e1006685. doi: 10.1371/journal.pcbi.1006685 (PMC6363221; doi:10.1371/journal.pcbi.1006685)
Supplement: S2 File — Also includes: Table S1 Model species. The names, description, and initial amounts (particle numbers) of all species in the complete model. Table S2 Model reactions. The reactions and parameter values in the complete model. (PDF) [file pcbi.1006685.s013.pdf]

Systems biology reveals how altered TGFβ signalling with age reduces protection against pro-inflammatory stimuli

David Hodgson[1,4], Andrew D. Rowan[2,4], Francesco Falciani[3,4], Carole J. Proctor[1,4*]

1 Institute of Cellular Medicine, Ageing Research Laboratories, Campus for Ageing and Vitality, Newcastle University, Newcastle upon Tyne, UK. **2** Skeletal Research Group, Institute of Genetic Medicine, Newcastle University, Newcastle upon Tyne, UK. **3** Institute of Integrative Biology, Biosciences Building, University of Liverpool, Liverpool, UK. **4** MRC/Arthritis Research UK Centre for Musculoskeletal Ageing (CIMA), UK

* Corresponding author

Supplementary details of model construction

The complete model schematic can be seen in S5 Fig, and is a modified expansion of two previously published models [1,2]. These were simplified in ordered to reduce computational burden whilst still fitting the same profiles shown in the original models (S9-11 Figs). In order to achieve this, some assumptions were made which marginally changed the structures. The original IL-1+OSM model was presented in three sections, OSM, IL-1 and pro-MMP activation [1]. As we were only looking at *MMP13* mRNA, and did not explore the transition to active MMP, we removed the pro-MMP activation component completely. The remaining two sections were simplified ensuring that they still fitted the same profiles. To achieve this some approximations were made. Three proteins were originally present in the model: Dusp16, Mkp1 and Pp4. As these were upregulated by the same source and only relevant for blocking, we replaced all three of these species with one "Block" species. This blocked all three reactions that the original proteins did. However, each reaction was blocked at a different rate in order to have the same effect that the original proteins would have had. In the original model Traf6 was internalised by Irak2 binding to receptor bound IL-1. We changed the receptor profile for IL-1 to include an internalisation step that replaced the need for Irak2 and Traf6. The phosphorylation and subsequent nuclear translocation of Stat3 was also altered so that it could be represented by a single self-limiting reaction. Finally, the original models included mRNAs for many of the proteins. Apart from cFos and *MMP13*, these were removed so that protein synthesis could be more simply modelled by one reaction.

The Alk1/Alk5 sections of the model are based on the TGFβ section described in [2]. The most significant change was the removal of the Sox9, aggrecan and collagen components from the original model as these were not of interest in the current study. Mmp2 was completely removed as sensitivity analysis showed it had no effect on the model in this form. The role of active Runx2 was also altered, no longer inducing the production of pro-MMP-13 but instead producing *MMP13* mRNA. This change was reasonable as Runx2-induced upregulation of *MMP13* has been shown to be at the mRNA level [3]. We also included an additional reaction so that Smad1/5/8 signalling leads to an increase in p38 phosphorylation [4].

The original models had not been created to be combined and as a result they did not share any interactions. Therefore, the combined model was extended to include reactions that link the TGFβ and IL-1+OSM signalling pathways, based on knowledge

from the literature. We considered two different mechanisms: inhibition of AP-1 (cJun homodimers or cJun/cFos heterodimers) by JunB; and increased degradation of *MMP13* mRNA via TGFβ signalling (see below).

Inhibition of AP-1 by JunB

There is a substantial body of evidence showing that JunB is a direct downstream target of Smad3 [5,6] and serves to antagonise rapid gene transcription [7-9]. Studies have shown it can displace cFos in the AP-1 complex and bind to cJun [5,7]. It has also been demonstrated to limit the effects of IL-1, in particular its effect on MMP synthesis [10]. Finally, it has been shown to repress gene expression in epithelial-mesenchymal transition in cancer due to upregulation by TGFβ. This demonstrates TGFβ can induce significantly high JunB expression to limit gene transcription [6]. We modelled these effects by adding reactions for JunB production via Smad2/3 and JunB binding to cJun to inhibit AP-1 transcription (see yellow highlighted cells in Table 2).

Increased degradation of *MMP13* mRNA via Smad2/3

As the protein or microRNA responsible for increased *MMP13* mRNA degradation via Smad2/3 signalling is currently unknown we added a species with ID 'Dummy'. We assumed that transcription of the 'Dummy' gene requires an activator protein 'A' and that 'A' needs to be phosphorylated by Smad2/3 for its activity. The 'Dummy' protein then interacts with *MMP13* mRNA to induce its degradation. The reactions for this mechanism are shown by the highlighted green cells in Table 2.

Model simplifications

There were some assumptions still in place from the original models that should be mentioned. These were justified in the supplementary data in [2] and the main text of [1]. The first model simplification is that TGFβ turnover is not included, and so it can only be added to the model in the initial conditions or via an event. This simplification was left in place as TGFβ can enter the system for a variety of reasons and incorporating these into the model would have added significant complexity. Instead, TGFβ is inactivated and activated in the model but the total pool remains constant. Any active TGFβ that enters the system is inactivated in a process that degrades Alk5 and Smad7. Alk1 can also be degraded if TGFβ is bound to an Alk1/Alk5 heterodimer. Inactive TGFβ can then be activated by integrin, which was included as a species in the model. It is known that TGFβ can be activated by many biological components such as plasmin, F-spondin or MMP-2/-9 [11,12] as well natural detergents such as lysophosphatidylethanolamine (LPE) and lysophosphatidylcholine (LPC) [13]. For simplification, Integrin is representative of all mechanisms of TGFβ activation.

The TGFβ-driven increase in *MMP13* mRNA is by Runx2, which is activated by phospho-Smad1/5/8 and inactivated by phospho-Smad2/3. This is a simplification as Runx2 can be inactivated by other mechanisms [14]. However, as we are specifically interested in how TGFβ affects Runx2, having just phospho-Smads affecting its activation provides insight into how their effect on Runx2 changes with age.

The model works through the assumption that IL-1+OSM-driven *MMP13* expression is a result of cFos/cJun heterodimers and cJun homodimers. Our group has recently reported that this may be a simplistic view since ATF3 appears to be important in this upregulation [15]. However, it is also evident that cFos/cJun heterodimers have a significant effect on ATF3 expression [15], such that it is reasonable to assume that blocking cFos/cJun in the way predicted in the model would have the same overall effect on *MMP13* expression.

The combined model uses mass action kinetics but there are differences in the kinetic rate laws for homodimer formation between the deterministic and stochastic versions. In the deterministic model, the rate law for a homodimerisation reaction, $2X \rightarrow Y$ is $k*X^2$. However, in stochastic models, the rate is proportional to the number of ways that two molecules of type X can interact, namely X*(X-1)/2. This means that the reaction can only take place if there are at least two molecules of X. An example is for cJun dimerization which has the equation: *k1**(cJun_P)*(cJun_P-1)*0.5. Only one compartment was used for the model entitled "cell". The complete lists of species and reactions in the combined model are shown in Tables S1 and S2.

Further details of parameter estimation

To parametrise the model we initially ran a global parameter scan using the genetic algorithm, followed immediately by a local algorithm Hooke and Jeeves. To fit *MMP13* data we used the percentage of repression in the IL-1+OSM+TGFβ samples, compared to IL-1+OSM alone. This was accomplished by simulating the model with only IL-1+OSM active, then using the generated data to determine the level of repression necessary at each time point. For example, at 24 hours the particle number for *MMP13* with simulated IL-1+OSM treatment was 109. We knew from the data that when TGFβ was present this should be reduced by 42%, so at 24 hours the value was changed to 63. This was repeated for all known time points and the IL-1+OSM+TGFβ model was fitted to those data.

Table A. Model species. The IDs, description, and initial amounts of all species in the complete model.

Species	Description	Initial amount (particle number)
IL1	IL-1	100
Jnk_P	Phosphorylated form of Jnk	0
Jnk	Jnk	100
IL1R	IL-1 receptor	100
IL1RR	IL-1 bound to receptor	0
IL1RR.int	IL-1 bound to receptor and internalised	0
cJun	cJun	100
cJun_P	Phosphorylated form of cJun	0
cJun_dimer	cJun dimer	0
OSM	OSM	1000
OSMR	OSM receptor	100
OSMRR	OSM bound to receptor	0
Jak1	Jak1	100
Jak1_P	Phosphorylated form of Jak1	0
Stat3_nuc	Stat3 in the nucleus	0
cFos_mRNA	cFos mRNA	0
Stat3_cyt	Stat3 in the cytoplasm	75
cFos	cFos	0
p38	p38	100
p38_P	Phosphorylated form of p38	0
cFos_P	Phosphorylated form of cFos	0

cFos_cJun	cFos/cJun heterodimer	0
Block	A dummy species to represent 3 proteins that inhibit signalling	0
MMP13_mRNA	MMP-13 mRNA	0
Integrin	Integrin but also representative of all mechanisms of TGFβ activation	0
Source (fixed particle number)	A fixed pool for the creation of new molecules	1
Sink (fixed particle number)	A fixed pool for the degradation of molecules	1
Alk5	Alk5	500
Tgfb_A	Active form of TGFβ	0
Tgfb_I	Inactive form of TGFβ	200
Alk5_dimer	Alk5 homodimer	0
Alk1	Alk1	300
Alk1_Alk5	Alk1/Alk5 heterodimer	0
Tgfb_Alk5_dimer	Active TGFβ bound to an Alk5 homodimer	0
Smad7	Smad7	0
Tgfb_Alk5_dimer_Smad7	Active TGFβ bound to an Alk5 homodimer and Smad7	0
Tgfb_Alk1_Alk5	Active TGFβ bound to an Alk1/Alk5 heterodimer	0
Smad2_3	Smad2/3	600
Smad2_3_P	Phosphorylated form of Smad2/3	0
Smad2_3_P_Smad4	Phosphorylated form of Smad2/3 bound to Smad4	0
Smad4	Smad4	600
Runx2_A	Active form of Runx2	0
Runx2_I	Inactive form of Runx2	100
Smad1_5_8	Smad1/5/8	600
Smad1_5_8_P	Phosphorylated form of Smad1/5/8	0
Smad1_5_8_P_Smad4	Phosphorylated form of Smad1/5/8 bound to Smad4	0
Tgfb_Alk1_Alk5_Smad7	Active TGFβ bound to an Alk1/Alk5 heterodimer and Smad7	0
JunB_mRNA	JunB mRNA	0
JunB	JunB	0
JunB_cJun	JunB bound to cJun	0
Dummy	A dummy species which induces MMP-13 mRNA degradation	0
Dummy_mRNA	Dummy species mRNA	0
A	Protein needed to drive dummy production	100
A_P	Protein needed to drive dummy production phosphorylated	0

Table B. Model reactions. The reactions and parameter values in the complete model. Yellow highlighted cells indicate reactions for inhibition of AP-1 by JunB; green highlighted cells indicate reactions for *MMP13* mRNA degradation via Smad2/3 pathway.

Reactions	Parameter values
IL1 ⟶ sink	0.0134 s^{-1}
Jnk + IL1RR.int ⟶ Jnk_P + IL1RR.int	7.07E-05 mol^{-1} s^{-1}
Jnk_P ⟶ Jnk	1.17E-03 s^{-1}
IL1 + IL1R ⟶ IL1RR	1.56E-05 mol^{-1} s^{-1}
IL1RR ⟶ IL1 + IL1R	4.56E-04 s^{-1}
IL1RR ⟶ IL1RR.int	5.26E-04 s^{-1}
IL1RR.int ⟶ IL1RR	1.09E-04 s^{-1}
cJun + Jnk_P ⟶ cJun_P + Jnk_P	5.18E-04 mol^{-1} s^{-1}
cJun_P ⟶ cJun	0.0101 s^{-1}
2 * cJun_P ⟶ cJun_dimer	3.46E-05 mol^{-1} s^{-1}
cJun_dimer ⟶ 2 * cJun_P	5.98E-03 s^{-1}
OSM + OSMR ⟶ OSMRR	5.65E-06 mol^{-1} s^{-1}
OSMRR ⟶ OSM + OSMR	1.46E-04 s^{-1}
OSM ⟶ Sink	4.90E-03 s^{-1}
Jak1 + OSMRR ⟶ Jak1_P + OSMRR	1.15E-05 mol^{-1} s^{-1}
Stat3_cyt + Jak1_P ⟶ Stat3_nuc + Jak1_P	2.55E-04 mol^{-1} s^{-1}
Jak1_P ⟶ Jak1	1.13E-04 s^{-1}
Stat3_nuc ⟶ Stat3_cyt	5.17E-03 s^{-1}
cFos_mRNA ⟶ Sink	1.93E-03 s^{-1}
Stat3_nuc ⟶ Stat3_nuc + cFos_mRNA	0.0373 s^{-1}
Stat3_nuc + Jak1_P ⟶ Jak1 + Stat3_nuc	2.52E-06 mol^{-1} s^{-1}
cFos_mRNA ⟶ cFos_mRNA + cFos	6.38E-04 s^{-1}
cFos ⟶ Sink	8.93E-05 s^{-1}
p38 + IL1RR.int ⟶ p38_p + IL1RR.int	1.03E-04 mol^{-1} s^{-1}
p38_P ⟶ p38	3.75E-04 s^{-1}
p38_P + cFos ⟶ p38_P + cFos_P	1.23E-05 mol^{-1} s^{-1}
cFos_P ⟶ cFos	0.0249 s^{-1}
cFos_P + cJun_P ⟶ cFos_cJun	5.17E-05 mol^{-1} s^{-1}
cFos_cJun ⟶ cFos_P + cJun_P	3.81E-05 s^{-1}
cJun_dimer ⟶ cJun_dimer + Block	2.09E-04 s^{-1}

cFos_cJun ⟶ cFos_cJun + Block	4.18E-03 s^{-1}
Block ⟶ Sink	1.82E-05 s^{-1}
Block + IL1RR.int ⟶ Block + IL1R	1.81E-04 mol^{-1} s^{-1}
Jnk_P + Block ⟶ Jnk + Block	3.51E-04 mol^{-1} s^{-1}
Stat3_nuc + Block ⟶ Stat3_cyt + Block	2.91E-03 mol^{-1} s^{-1}
cFos_P + Block ⟶ cFos + Block	6.58E-05 mol^{-1} s^{-1}
cJun_dimer ⟶ MMP13_mRNA + cJun_dimer	1.54E-03 s^{-1}
cFos_cJun ⟶ MMP13_mRNA + cFos_cJun	4.75E-04 s^{-1}
MMP13_mRNA ⟶ Sink	3.48E-06 s^{-1}
Source ⟶ Integrin	2.26E-07 mol^{-1} s^{-1}
Integrin ⟶ Sink	5.09E-04 s^{-1}
Source ⟶ Alk5	2.59E-06 mol^{-1} s^{-1}
Tgfb_I + Integrin ⟶ Tgfb_A + Integrin	8.32E-04 mol^{-1} s^{-1}
Tgfb_A ⟶ Tgfb_I	0.0309 s^{-1}
2 * Alk5 ⟶ Alk5_dimer	1.19E-04 mol^{-1} s^{-1}
Alk5_dimer ⟶ 2 * Alk5	7.43E-04 s^{-1}
Alk1 + Alk5 ⟶ Alk1_Alk5	7.65E-05 mol^{-1} s^{-1}
Alk1_Alk5 ⟶ Alk1 + Alk5	0.0105 s^{-1}
Tgfb_A + Alk5_dimer ⟶ Tgfb_Alk5_dimer	3.54E-05 mol^{-1} s^{-1}
Tgfb_Alk5_dimer ⟶ Tgfb_A + Alk5_dimer	1.11E-06 s^{-1}
Tgfb_Alk5_dimer + Smad7 ⟶ Tgfb_Alk5_dimer_Smad7	1.65E-05 mol^{-1} s^{-1}
Tgfb_Alk5_dimer_Smad7 ⟶ Tgfb_Alk5_dimer + Smad7	1.64E-06 s^{-1}
Tgfb_Alk5_dimer_Smad7 ⟶ Tgfb_I	2.06E-05 s^{-1}
Tgfb_A + Alk1_Alk5 ⟶ Tgfb_Alk1_Alk5	4.77E-06 mol^{-1} s^{-1}
Tgfb_Alk1_Alk5 ⟶ Tgfb_A + Alk1_Alk5	1.56E-06
Tgfb_Alk5_dimer + Smad2_3 ⟶ Tgfb_Alk5_dimer + Smad2_3_P	3.12E-05 mol^{-1} s^{-1}
Smad2_3_P + Smad4 ⟶ Smad2_3_P_Smad4	5.30E-05 mol^{-1} s^{-1}
Smad2_3_P_Smad4 ⟶ Smad2_3_P + Smad4	0.0109 s^{-1}
Smad2_3_P ⟶ Smad2_3	8.80E-03 s^{-1}

Smad2_3_P_Smad4 ⟶ Smad2_3_P_Smad4 + Smad7	8.94E-06 s^{-1}
Runx2_A + Smad2_3_P_Smad4 ⟶ Runx2_I + Smad2_3_P_Smad4	7.17E-04 mol^{-1} s^{-1}
Alk5 ⟶ Sink	3.45E-07 s^{-1}
Tgfb_Alk1_Alk5 + Smad1_5_8 ⟶ Tgfb_Alk1_Alk5 + Smad1_5_8_P	1.69E-05 mol^{-1} s^{-1}
Smad1_5_8_P ⟶ Smad1_5_8	2.17E-04 s^{-1}
Smad1_5_8_P + Smad7 ⟶ Smad1_5_8 + Smad7	3.77E-04 mol^{-1} s^{-1}
Smad1_5_8_P + Smad4 ⟶ Smad1_5_8_P_Smad4	4.70E-05 mol^{-1} s^{-1}
Smad1_5_8_P_Smad4 ⟶ Smad1_5_8_P + Smad4	0.0177 s^{-1}
Runx2_I + Smad1_5_8_P_Smad4 ⟶ Runx2_A + Smad1_5_8_P_Smad4	1.66E-03 mol^{-1} s^{-1}
Runx2_A ⟶ MMP13_mRNA + Runx2_A	1.59E-06 s^{-1}
Source ⟶ Alk1	4.88E-06 mol^{-1} s^{-1}
Alk1 ⟶ Sink	1.70E-08 s^{-1}
Tgfb_Alk1_Alk5 + Smad7 ⟶ Tgfb_Alk1_Alk5_Smad7	0.265 mol^{-1} s^{-1}
Tgfb_Alk1_Alk5_Smad7 ⟶ Tgfb_Alk1_Alk5 + Smad7	7.60E-04 s^{-1}
Tgfb_Alk1_Alk5_Smad7 ⟶ Tgfb_I	9.77E-06 s^{-1}
Smad7 ⟶ Sink	7.97E-03 s^{-1}
Smad2_3_P_Smad4 ⟶ Smad2_3_P_Smad4 + JunB_mRNA	1.71E-05 s^{-1}
p38 + Smad1_5_8_P_Smad4 ⟶ p38_P + Smad1_5_8_P_Smad4	1.90E-05 mol^{-1} s^{-1}
JunB_mRNA ⟶ JunB_mRNA + JunB	8.52E-04 s^{-1}
JunB_mRNA ⟶ Sink	1.48E-05 s^{-1}
JunB ⟶ Sink	9.24E-03 s^{-1}
JunB + cJun_dimer ⟶ cJun_P + JunB_cJun	1.38E-06 mol^{-1} s^{-1}
JunB + cJun_P ⟶ JunB_cJun	6.97E-04 mol^{-1} s^{-1}
JunB_cJun ⟶ JunB + cJun_P	3.37E-05 s^{-1}
cFos_cJun + JunB ⟶ JunB_cJun + cFos_P	6.75E-06 mol^{-1} s^{-1}

Smad2_3_P_Smad4 ⟶ Smad2_3_P_Smad4 + A_P	1.42E-05 s^{-1}
A_P ⟶ Dummy_mRNA + A_P	1.46E-05 s^{-1}
Dummy_mRNA ⟶ Dummy	1.54E-05 s^{-1}
Dummy + MMP13_mRNA ⟶ Dummy	1.07E-06 mol^{-1} s^{-1}
Dummy_mRNA ⟶ Sink	3.86E-05 s^{-1}
Dummy ⟶ Sink	3.22E-05 s^{-1}
A_P ⟶ A	3.79E-04 s^{-1}

References

1. Proctor CJ, Macdonald C, Milner JM, Rowan AD, Cawston TE. A computer simulation approach to assessing therapeutic intervention points for the prevention of cytokine-induced cartilage breakdown. Arthritis Rheumatol. 2014; 66: 979-989.
2. Hui W, Young DA, Rowan AD, Xu X, Cawston TE, Proctor CJ. Oxidative changes and signalling pathways are pivotal in initiating age-related changes in articular cartilage. Ann. Rheum. Dis. 2016; 75: 449-458.
3. Ijiri K, Zerbini LF, Peng H, Correa RG, Lu B, Walsh N, et al. A novel role for GADD45β as a mediator of MMP-13 gene expression during chondrocyte terminal differentiation. J. Biol. Chem. 2005; 280: 38544-38555.
4. Chen CG, Thuillier D, Chin EN, Alliston T. Chondrocyte-intrinsic Smad3 represses Runx2-inducible matrix metalloproteinase 13 expression to maintain articular cartilage and prevent osteoarthritis. Arthritis Rheumatol. 2012; 64: 3278-3289.
5. Ponticos M, Harvey C, Ikeda T, Abraham D, Bou-Gharios G. JunB mediates enhancer/promoter activity of COL1A2 following TGF-beta induction. Nucleic Acids Res. 2009; 37: 5378-5389.
6. Gervasi M, Bianchi-Smiraglia A, Cummings M, Zheng Q, Wang D, Liu S, et al. JunB contributes to Id2 repression and the epithelial-mesenchymal transition in response to transforming growth factor-beta. J. Cell Biol. 2012; 196: 589-603.
7. Mauviel A, Chung KY, Agarwal A, Tamai K, Uitto J. Cell-specific induction of distinct oncogenes of the Jun family is responsible for differential regulation of collagenase gene expression by transforming growth factor-beta in fibroblasts and keratinocytes. J. Biol. Chem. 1996; 271: 10917-10923.
8. Verrecchia F, Tacheau C, Schorpp-Kistner M, Angel P, Mauviel A. Induction of the AP-1 members c-Jun and JunB by TGF-beta/Smad suppresses early Smad-driven gene activation. Oncogene. 2001; 20: 2205-2211.
9. Selvamurugan N, Kwok S, Partridge NC. Smad3 interacts with JunB and Cbfa1/Runx2 for transforming growth factor-beta1-stimulated collagenase-3 expression in human breast cancer cells. J. Biol. Chem. 2004; 279: 27764-27773.
10. Shimizu E, Selvamurugan N, Westendorf JJ, Olson EN, Partridge NC. HDAC4 represses matrix metalloproteinase-13 transcription in osteoblastic cells, and parathyroid hormone controls this repression. J. Biol. Chem. 2010; 285: 9616-9626.

11. Yu Q, Stamenkovic I. Cell surface-localized matrix metalloproteinase-9 proteolytically activates TGF-beta and promotes tumor invasion and angiogenesis. Genes Dev. 2000; 14: 163-176.
12. Attur MG, Palmer GD, Al-Mussawir HE, Dave M, Teixeira CC, Rifkin DB, et al. F-spondin, a neuroregulatory protein, is up-regulated in osteoarthritis and regulates cartilage metabolism via TGF-beta activation. FASEB J. 2009; 23: 79-89.
13. Gay I, Schwartz Z, Sylvia VL, Boyan BD. Lysophospholipid regulates release and activation of latent TGF-beta1 from chondrocyte extracellular matrix. Biochim. Biophys. Acta. 2004; 1684: 18-28.
14. Jonason JH, Xiao G, Zhang M, Xing L, Chen D. Post-translational regulation of Runx2 in bone and cartilage. J. Dent. Res. 2009; 88: 693-703.
15. Chan CM, Macdonald CD, Litherland GJ, Wilkinson DJ, Skelton A, Europe-Finner GN, et al. Cytokine-induced MMP13 expression in human chondrocytes is dependent on activating transcription factor 3 (ATF3) regulation. J. Biol. Chem. 2017; 292: 1625-1636.
